# Methods for Evaluating Respondent Attrition in Web-Based Surveys

**DOI:** 10.2196/jmir.6342

**Published:** 2016-11-22

**Authors:** Camille J Hochheimer, Roy T Sabo, Alex H Krist, Teresa Day, John Cyrus, Steven H Woolf

**Affiliations:** ^1^Department of BiostatisticsVirginia Commonwealth UniversityRichmond, VAUnited States; ^2^Department of Family Medicine and Population HealthVirginia Commonwealth UniversityRichmond, VAUnited States; ^3^Tompkins-McCaw LibraryVirginia Commonwealth UniversityRichmond, VAUnited States

**Keywords:** patient dropouts, surveys and questionnaires, electronic health records

## Abstract

**Background:**

Electronic surveys are convenient, cost effective, and increasingly popular tools for collecting information. While the online platform allows researchers to recruit and enroll more participants, there is an increased risk of participant dropout in Web-based research. Often, these dropout trends are simply reported, adjusted for, or ignored altogether.

**Objective:**

To propose a conceptual framework that analyzes respondent attrition and demonstrates the utility of these methods with existing survey data.

**Methods:**

First, we suggest visualization of attrition trends using bar charts and survival curves. Next, we propose a generalized linear mixed model (GLMM) to detect or confirm significant attrition points. Finally, we suggest applications of existing statistical methods to investigate the effect of internal survey characteristics and patient characteristics on dropout. In order to apply this framework, we conducted a case study; a seventeen-item Informed Decision-Making (IDM) module addressing how and why patients make decisions about cancer screening.

**Results:**

Using the framework, we were able to find significant attrition points at Questions 4, 6, 7, and 9, and were also able to identify participant responses and characteristics associated with dropout at these points and overall.

**Conclusions:**

When these methods were applied to survey data, significant attrition trends were revealed, both visually and empirically, that can inspire researchers to investigate the factors associated with survey dropout, address whether survey completion is associated with health outcomes, and compare attrition patterns between groups. The framework can be used to extract information beyond simple responses, can be useful during survey development, and can help determine the external validity of survey results.

## Introduction

### Background

Web-based surveys are convenient and cost-effective means for collecting research information. Researchers can reach a large number of participants quickly through electronic media, such as email and websites, when compared with conventional paper-based surveys. Applications like REDCap (REDCap Consortium) and SurveyMonkey (SurveyMonkey, Inc) automate the data collection and storage process as well as provide the capability to capture survey paradata or metadata. Web-based paradata allow researchers to capture respondent actions in addition to responses and to track the time participants spend on particular questions [1]. By linking surveys to clinical databases such as electronic health records (EHR), participant characteristics and other information (eg, biomarkers, medical history, laboratory results) can be used to customize questions posed to respondents.

This technology’s relative ease in soliciting survey participants is coupled with an increased risk of survey attrition—participants dropping out. Potential respondents may ignore solicitations, whereas others may skip questions or exit the survey before answering all the questions. Proper testing before administration, such as completion of a principal component analysis or factor analysis of survey items [2,3], helps ensure the validity, internal consistency, and reliability of the proposed survey instrument. Although we encourage researchers to engage in formative research and test their survey to address the issue of attrition before administering a survey instrument, we have seen in other research and experienced firsthand that these measures are sometimes not enough to prevent attrition from occurring.

Attrition can occur through different mechanisms and produce different types of bias. Nonusage or nonresponse attrition occurs when participants are solicited but choose not to participate in a survey [4,5] and this has been studied extensively [6,7], whereas dropout attrition occurs when a participant begins a survey but does not complete it [4,5]. These 2 types of attrition also occur in randomized controlled trials (RCTs) and longitudinal studies conducted both in person and on the Web. Prior research in this area has investigated both the degree to which attrition from clinical trials occurs and methods for retaining participants [8-12]. In this paper, dropout attrition in the RCT setting and nonresponse attrition are not considered, as we are specifically interested in dropout attrition in surveys or questionnaires.

Respondent fatigue is another factor which leads to dropout attrition, especially when questions seem inappropriate or inapplicable [13-15]. Although subject attrition is an issue in all types of health services research, dropout in Web-based health research can exceed expectations, reducing statistical power and potentially introducing bias [4]. Additionally, dropout is often ill reported or presented in a way that prevents readers from being able to fully understand attrition [5]. In 2005, after observing a large proportion of dropouts in several eHealth interventions, Eysenbach called for a “science of attrition” and more appropriate models for reporting and analyzing this phenomenon [4]. This science has 2 facets: survey techniques for minimizing survey attrition and methods for analyzing attrition patterns within particular studies. This paper focuses on the latter. Survey attrition research has generally focused on nonresponse attrition—when those invited to complete a survey choose not to participate—and on ways to increase overall participation. When Christensen and Mackinnon called for better methods to model the patterns, causes, and consequences of attrition, Eysenbach added that authors should explicitly state attrition rates and analyze dropout whenever possible, providing insight into why and for whom the intervention or survey did or did not work [16,17]. The potential for electronically delivered surveys to capture detailed information beyond the survey responses make them ideal for these types of attrition analyses.

### Objectives

This paper discusses novel ways to measure and investigate “dropout attrition” [4] for online surveys. We propose a conceptual approach to analyze attrition that begins with visualizing where attrition occurs and is followed by identifying attrition trends or patterns and examining factors associated with attrition. The methods proposed here are not intended to be exhaustive, but rather to serve as a starting point for establishing the science of dropout attrition. The methods were illustrated through a Web-based survey administered to patients who were eligible or overdue for breast, colorectal, or prostate cancer screenings [18].

## Methods

### Methods for Evaluating Attrition

Our proposed approach for evaluating dropout attrition includes 3 steps that are as follows: (1) visualization, (2) confirmation, and (3) factor identification. These steps are arranged in the order of increasing thoroughness for investigating attrition, with each step providing a more nuanced and detailed picture. Thus, investigators can work through these steps as far as their needs require.

#### Visualizing Attrition

The graphic representation of participant dropout could help visualize attrition trends or patterns. We proposed 2 visualization types—bar charts and survival-type curves—each with several variations to highlight different attrition trends.

Bar charts that described the amount (proportion, percentage, or number) of respondents or dropouts for each survey item provided multiple perspectives to explore dropout patterns. They allowed identification of differences between sequential questions, isolation of questions of specific interest, and discovery of overall trends. Plotting the percentage or proportion of respondents or dropouts was useful for identifying potentially significant attrition trends. Whether one plots respondents or dropouts depends on personal interest, although these might not be the exact inverses if the survey allows respondents to skip items. Plotting the raw number of dropouts was useful for finding other points of attrition that were not obvious when plotting proportions. Although not statistically significant, these trends provided information about when respondents left the survey, information that could be useful while testing a new survey instrument. Further, the stacked bar chart, which added the percentage of skips in each question, helped to better visualize attrition for surveys with skip patterns. Grouped bar charts were useful for comparing attrition visually between groups. As these final 2 types of bar charts may not be applicable, we suggest, at minimum, plotting the percentage of respondents or dropouts, along with the raw number of dropouts, to visualize attrition patterns.

Survival-type curves (or step functions) provided another way to visualize attrition. Unlike traditional survival curves, which stipulate decreasing patterns, these plots could incorporate situations in which the number of responses increased (eg, when a large number of respondents skip a particular item). These plots provided visual comparison of several groups with more clarity than the grouped bar chart, especially when comparing more than 3 groups. This visualization type was also useful for identifying what Eysenbach describes as the sigmoidal attrition curve, a pattern that includes a “curiosity plateau” at the beginning of the survey when response rates are high, an attrition phase when response rates decrease, and a stable participation phase when response rates are relatively constant for the remainder of the survey [4].

#### Confirming Significant Attrition

The second step was to determine whether any visually identified attrition patterns were statistically significant. A statistical model could determine the attrition changes from question to question. For example, a generalized linear mixed model (GLMM)—a broad set of models that includes logistic and Poisson regression—could incorporate both fixed and random effects to test if the proportion of patient responses decreases between subsequent questions [19]. Unlike simpler approaches (such as a chi-square test), GLMMs can account for the subject-level dependence due to previous attrition, which determines whether a subject responds to subsequent questions.

We applied a GLMM to test the hypothesis that the proportion of respondents is equal between 2 sequential questions. In our model, the outcome is binary, whether or not a person answered the survey questions (yes or no). An indicator for identifying the previous or subsequent question was included as a fixed effect and a subject-level random effect was included to account for within-subject dependence between response rates. The GLIMMIX procedure in the SAS software (SAS Institute) can be used to fit the GLMM to each pair of sequential questions. To transform the results into the difference in proportions, the IML procedure is needed to apply the multivariate delta method and thereby obtain a point estimate of the difference in response rates, along with the standard error and 95% CI for each comparison. The NLMIXED procedure could also be used to directly obtain point estimates of the difference in response rates between subsequent questions, but it does not allow for the covariance structure necessary to model more than 2 questions at a time.

#### Identifying Respondent Factors Associated With Attrition

The final step was to examine different factors that may be associated with attrition, such as patient characteristics (eg, age, gender), health outcomes (eg, cancer screening), survey responses, and survey metadata. Knowing that significant attrition trends exist in the dataset, we investigated factors associated with the observed dropout; high attrition rates could be attributable to any number of factors, including the survey itself. Results could also be stratified by population subgroups, such as gender, race, and ethnicity. In addition to looking at attrition question by question, we could also consider the overall attrition as a binary variable (ie, survey completers vs noncompleters).

We proposed 3 general methods for examining factors suspected to be associated with attrition: chi-square analyses (or Fisher’s exact test), the log-rank test, and Cox proportional hazards regression. Whereas previous research has used chi-square analyses to compare completers and noncompleters by demographics and lifestyle characteristics [20], we proposed the additional use of EHR data as well as survey characteristics, optimizing the use of an online platform to gather more information regarding attrition patterns.

We adopted Eysenbach’s suggestion for survival analysis [4] and used both the log-rank test and Cox proportional hazards regression to compare the overall trends in attrition. By comparing subsets of respondents, survival analysis helped us to verify that factors such as the language and content of the survey were not biased against particular groups. The log-rank test compared the overall attrition trends between mutually exclusive groups when survival trends were monotone (strictly decreasing). Cox proportional hazards regression was then used to adjust for other covariates that might confound or modify differences in survival trends; a significant covariate suggested confounding and a significant interaction suggested effect modification. For both the log-rank and Cox proportional hazards models, survival was defined as survey completion; respondents who completed the survey were deemed censored after the final question. This method has been previously used to compare groups in both the dropout attrition and nonusage attrition settings [20,21].

### Test Case

The survey—entitled the Informed Decision-Making (IDM) module—was designed by our research team to explore how people approach potentially difficult decisions about breast, colorectal, and prostate cancer screenings. It was developed in 2013 through intensive stakeholder engagement, including working with patients to ensure questions were in an understandable format that was easy to answer [18]. The survey consisted of 17 questions that explored patients’ awareness of cancer screening, chief concerns, and next steps [18]. Screenshots of these questions are provided in Multimedia Appendix 1. The IDM module also examined the patient’s agenda in discussing screening at their next appointment, including the format in which they preferred to receive information.

The study was conducted between January and August, 2014 at 12 primary care practices in northern Virginia that used the interactive online patient portal *MyPreventiveCare* (MPC) [22-25], which links directly to the practices’ EHR. The IDM module was programmed to query the EHR database to identify 3 groups of patients with MPC accounts: women aged 40-49 years who had not had a mammogram within 2 years, men aged 55-69 years who had not had a prostate-specific antigen test within 2 years, and adults aged 50-74 years who were not up-to-date with colorectal cancer screening. Those eligible for more than one screening test at the time of recruitment were invited to select which module they wanted to complete (see Multimedia Appendix 1). Patients were prompted to complete the IDM module during 3 distinct phases. In phase 1, patients meeting inclusion criteria were prompted to complete the module when using MPC for other reasons. During phase 2, eligible patients with an upcoming wellness visit were emailed up to 3 invitations to participate. In phase 3, every eligible patient in the practices’ EHR database, irrespective of whether they had a scheduled appointment, was emailed up to 3 invitations to complete the IDM module. Data for this study include patients’ responses to the IDM module supplemented with demographic information from the practices’ EHR.

Most questions in the IDM module had several subquestions. The system did not force respondents to answer all questions and allowed patients to skip questions. Five questions were directed to a subset of patients based on their answer to a previous question. Although these questions were imperative to our original study goals, we excluded them from this attrition analysis. The study was funded by the Patient Centered Outcomes Research Institute in 2012 and approved by the Virginia Commonwealth University Institutional Review Board [26].

All statistical analyses were conducted using SAS version 9.4 (SAS Institute), whereas all graphs were created using R version 3.1.1 (R Foundation for Statistical Computing) with the *rms, survival, ggplot2, gridExtra, rColorBrewer, and survminer* packages. Inferences were made at 5% significance level.

## Results

### Visualizing Attrition

**Figure 1:**
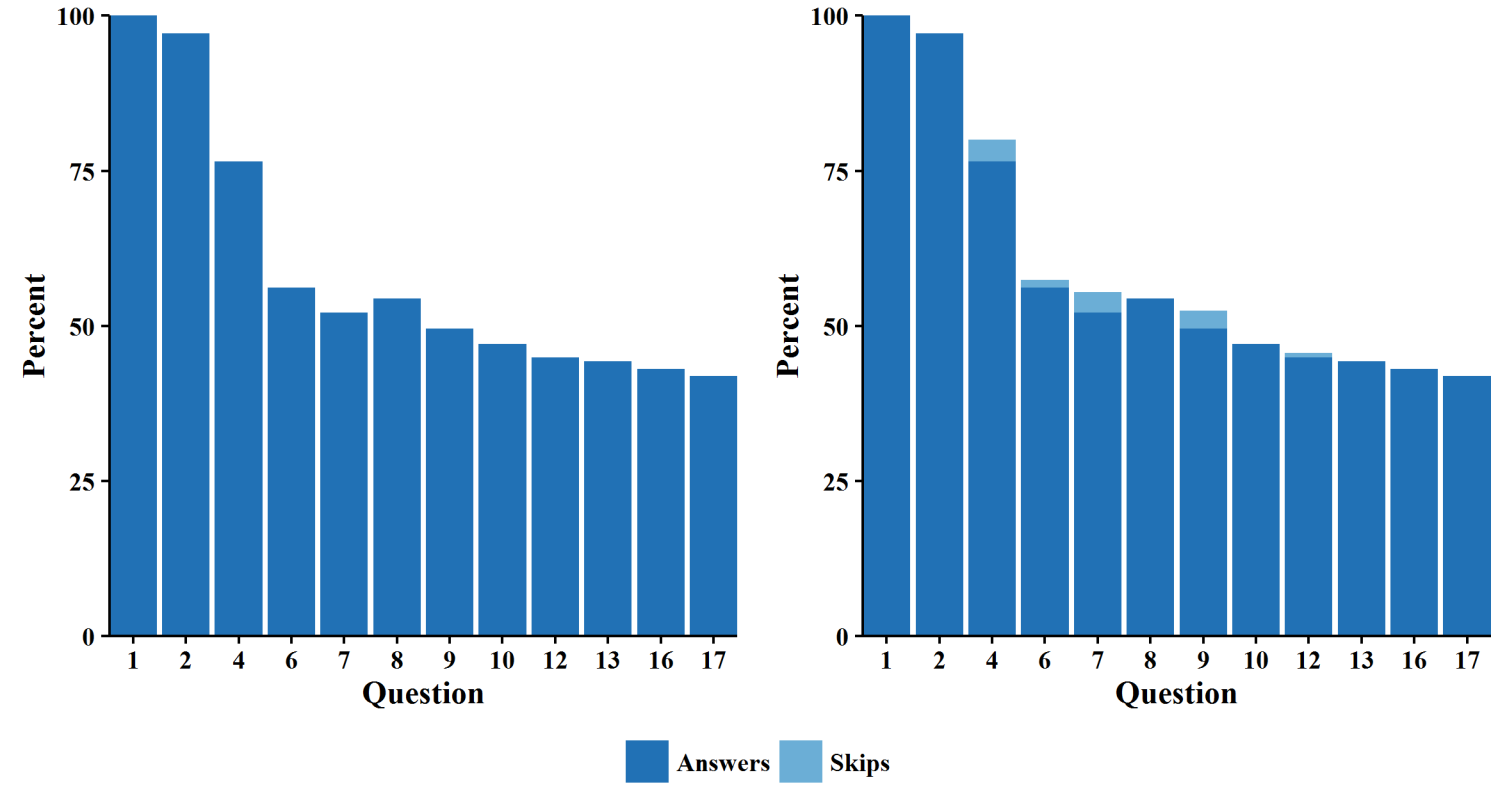
Bar charts for percent of answers for all cancer types without skips (left) and with skips (right).

During the study period, 2355 patients started the IDM module: 638 from the breast cancer cohort, 1249 from the colorectal cancer cohort, and 468 from the prostate cancer cohort. A bar chart displayed the percentage of respondents for each succeeding question in the module (Figure 1, left panel). It shows that the largest declines in the percentage of respondents occurred between Questions 2 and 4 and between Questions 4 and 6 (Questions 3 and 5 were ignored because they were directed only to specific subsets of subjects). After Question 6, the percentage of respondents remained relatively constant. Eysenbach’s curiosity plateau appeared to last until Question 2 [4]. The attrition phase began at Question 2 and ended after Question 6. This was followed by the stable participation phase, where the overall attrition rate converged to about 60%.

The bar chart reveals an increase in the percentage of patients who answered Question 8, which occurred because patients were able to skip questions. A stacked bar chart demonstrates that some participants skipped Questions 4, 6, 7, 9, and 12 (Figure 1, right panel).

**Figure 2:**
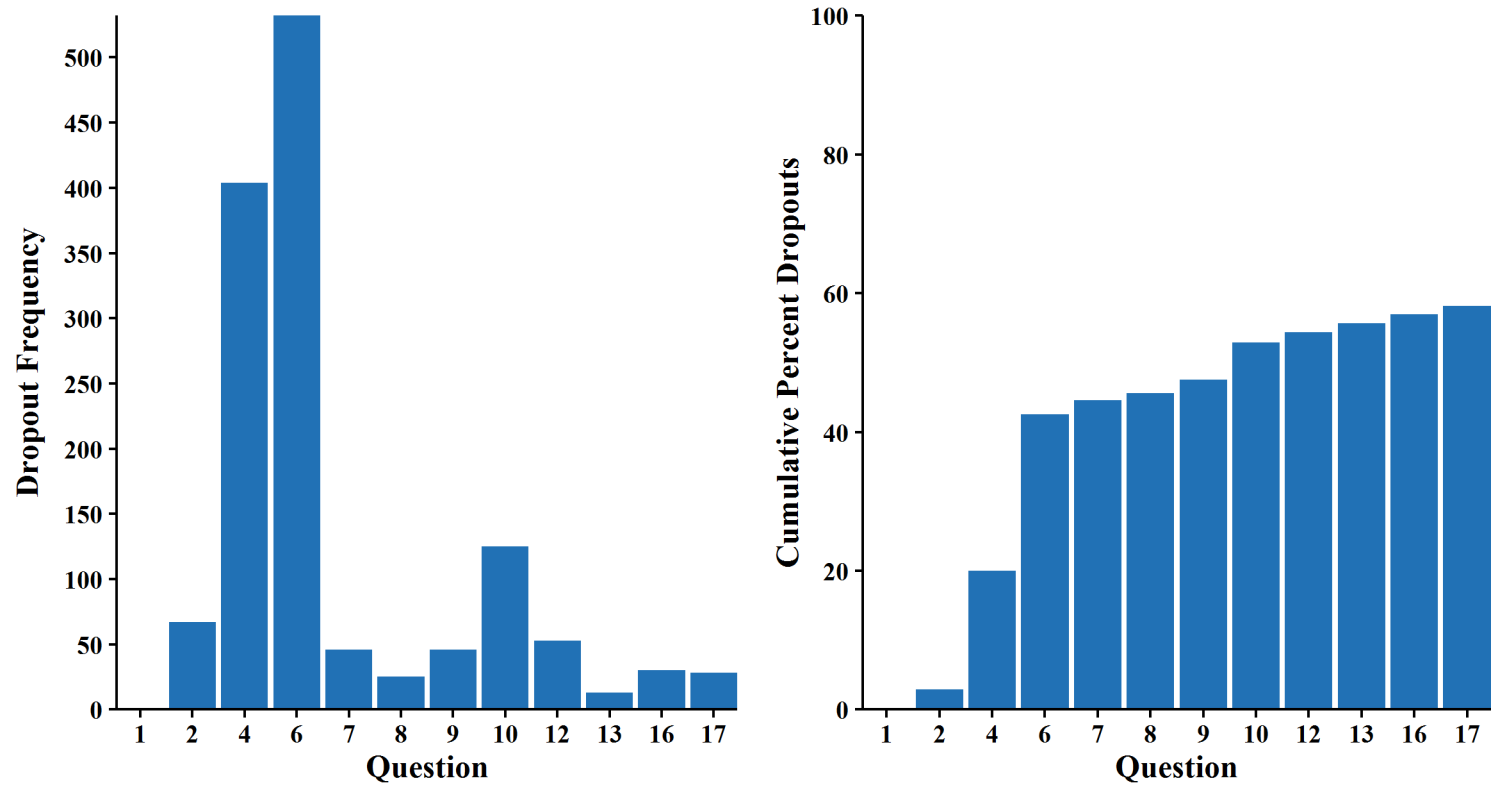
Bar charts for number of dropouts (left) and percent of dropouts (right).

The right panel of Figure 2, which plots the dropout rates for each question, again shows that the percentage of dropouts increased drastically between Questions 2 and 6, leveling off thereafter. The left panel of Figure 2, which plots the absolute number of dropouts by question, yet again shows that most attrition occurred at Questions 4 and 6 but also reveals a second wave of attrition around Question 10 that was not obvious in prior figures.

**Figure 3:**
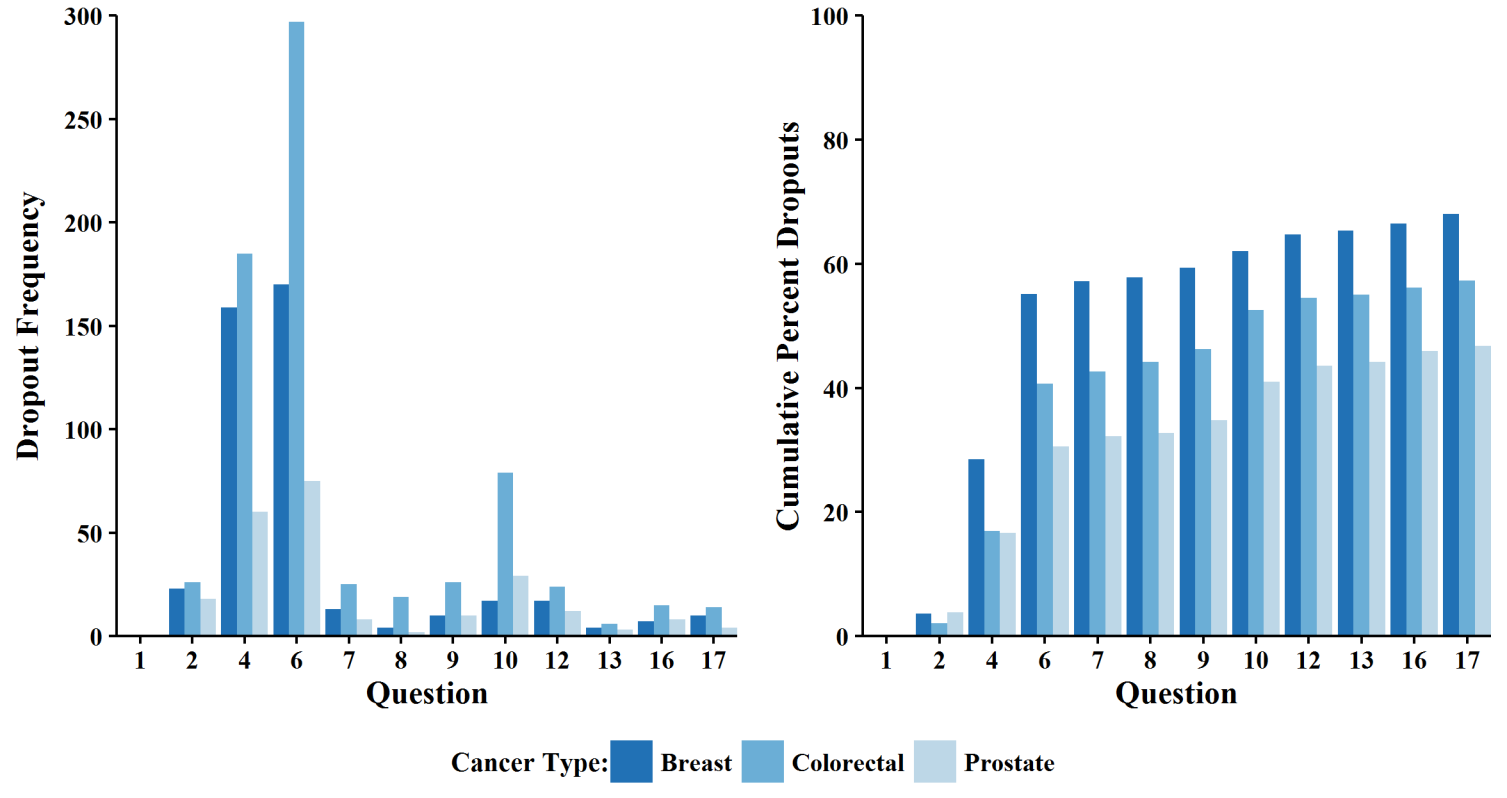
Grouped bar charts for the number of dropouts (left) and percent of dropouts (right).

We used grouped bar plots as per Ekman [1] to compare the number of dropouts by type of cancer screening (Figure 3, left). Whereas the general trends are consistent across cohorts, between-group comparisons are skewed due to the unequal sample sizes of each group (the colorectal cancer cohort was larger than both the breast and prostate cancer cohorts combined). Therefore, the right panel of Figure 3 compares the percentages of dropouts in each cohort with a grouped bar plot to adjust for differences in sample size. This plot shows that the breast cancer cohort had the highest attrition at each question while the prostate cancer cohort had the lowest attrition rate.

**Figure 4:**
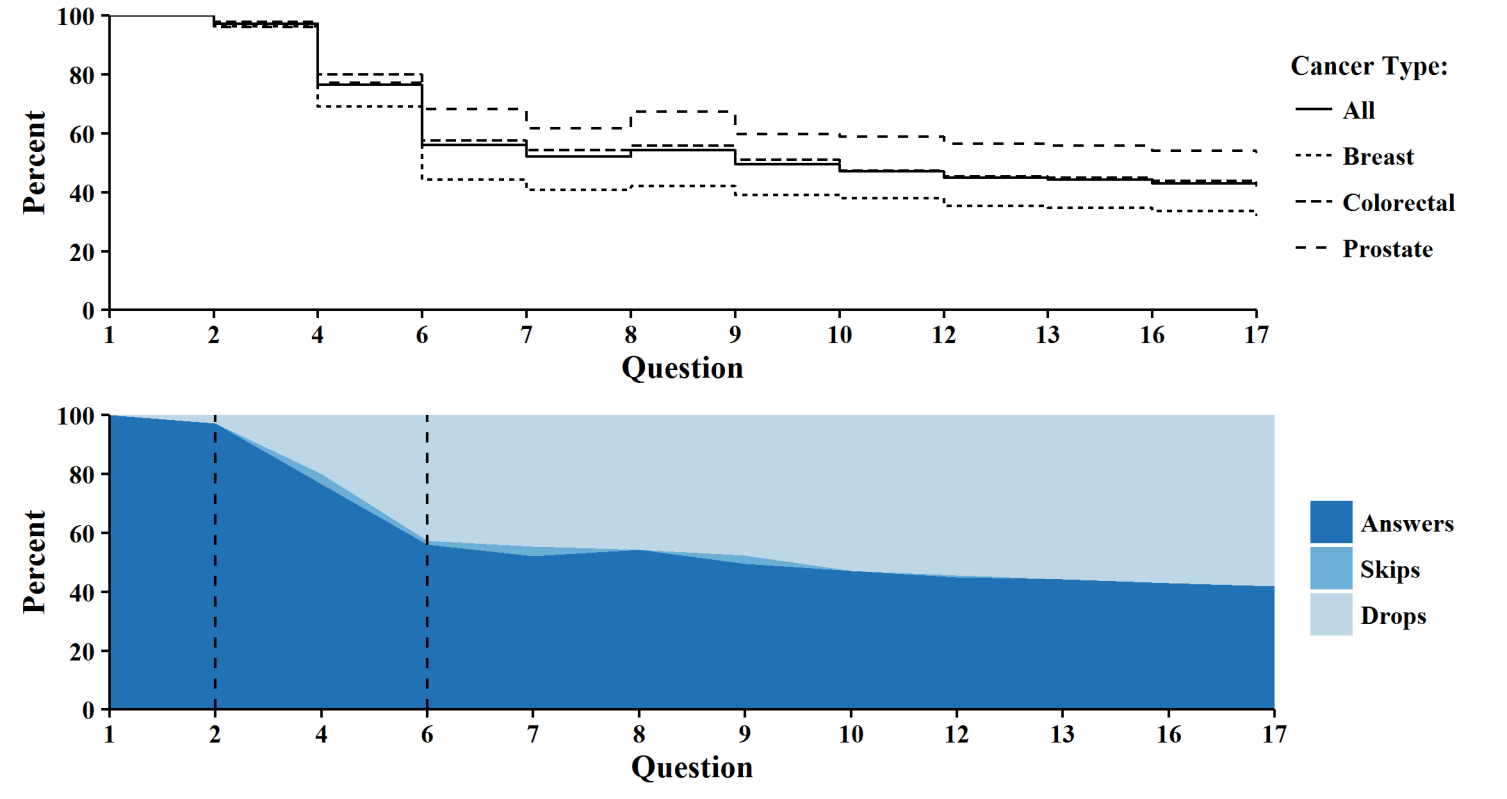
Step function comparing all cohorts (top) and attrition curve of all cancer types (bottom).

The top panel of Figure 4 displays the survival-like attrition curves for each cohort and overall, showing large vertical drops (ie, increased attrition) at Questions 4 and 6. This plot also highlights that the proportion of answers increased between Questions 7 and 8, a trend especially pronounced in the prostate cancer cohort. Overall dropout was highest in the breast cancer cohort and lowest in the prostate cancer cohort. The bottom panel of Figure 4 uses shading to display skips (as in the right panel of Figure 1) and vertical lines to highlight our estimation of the curiosity, attrition and stable phases per Eysenbach.

### Confirming Significant Attrition

As observed through visualization, the GLMM results suggest that the attrition that occurred between Questions 2 and 4, 4 and 6, 6 and 7, and 8 and 9 were statistically significant (*P*<.05, Table 1). These pairs of questions also exhibited the largest decreases in response rates (20.68%, 20.33%, 3.99%, and 4.88%, respectively). Between-question differences in response proportions were mostly positive, indicating that the response rates generally decreased (and attrition increased). An exception was the change in response rates between Questions 7 and 8 (52.14% and 54.39%, respectively), which increased and led to a negative difference (–2.25%). This pattern, observed visually in Figures 1 and 4, was due to some respondents skipping questions.

**Table 1:** Generalized linear mixed model (GLMM) results.

Analysis	p_1_	p_2_	p_1_-p_2_	Standard error	95% CI	*P* value
Q1 to Q2	1.00	0.97	0.03	0.660	−1.264 to 1.321	.99
Q2 to Q4	0.97	0.76	0.21	0.001	0.206 to 0.208	<.001
Q4 to Q6	0.76	0.56	0.20	0.001	0.203 to 0.204	<.001
Q6 to Q7	0.56	0.52	0.04	0.001	0.039 to 0.041	.006
Q7 to Q8	0.52	0.54	−0.02	0.001	−0.023 to −0.022	.12
Q8 to Q9	0.54	0.49	0.05	0.001	0.048 to 0.049	<.001
Q9 to Q10	0.49	0.47	0.02	0.001	0.023 to 0.024	.10
Q10 to Q12	0.47	0.45	0.02	0.001	0.022 to 0.023	.12
Q12 to Q13	0.45	0.44	0.01	0.001	0.005 to 0.006	.70
Q13 to Q16	0.44	0.43	0.01	0.001	0.012 to 0.013	.38
Q16 to Q17	0.43	0.42	0.01	0.001	0.011 to 0.012	.41

### Identifying Respondent Factors Associated With Attrition

We used the chi-square test to determine if a respondent’s answer to a particular question was associated with dropout in the next question and found that patients in the middle of the decision-making process—having indicated on Question 2 that they were either thinking about or close to making a decision (Multimedia Appendix 1)—were significantly less likely to drop out compared with those who had already made a choice or had not yet given the issue any thought (Table 2). Chi-square testing of subsequent screening behavior revealed that patients who completed the survey were more likely to get the screening test that their survey addressed than the noncompleters (22.37% and 17.42%, respectively, *P*=.003).

**Table 2:** Determining if a patient’s response to Question 2 (“How far along are you with making a decision about cancer screening?”) was associated with answering the next question.^a^

Response to Question 2	Answered Question 4	
	Yes (%)	No (%)
I have not yet thought about the choice.	79.47	20.53
I am thinking about the choice.	85.54	14.46
I am close to making a choice.	84.72	15.28
I have already made a choice.	75.24	24.76

^a^Overall chi-square test: *P*<.001

**Figure 5:**
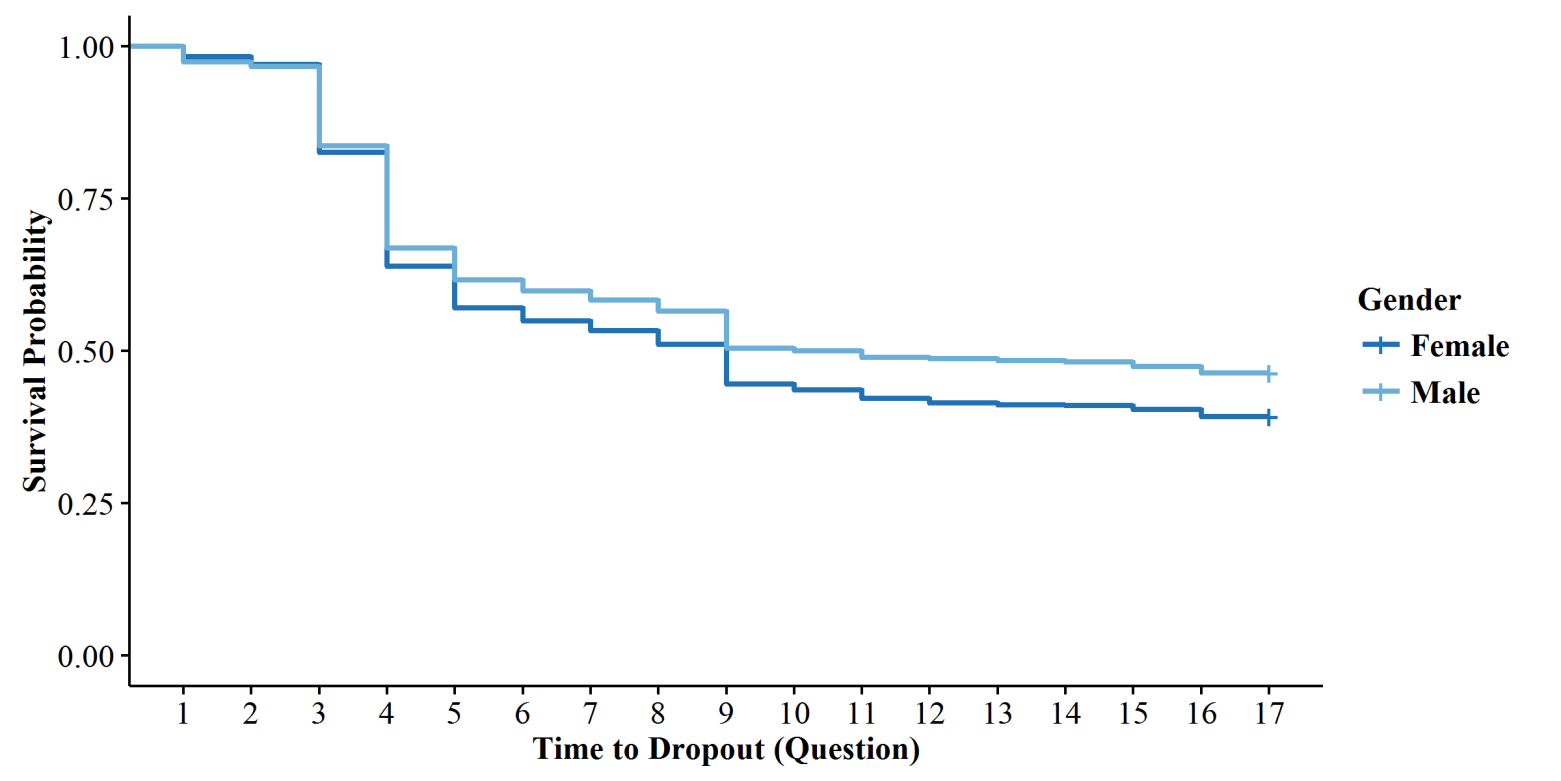
Kaplan-Meier survival curves by gender within colorectal cancer cohort.

We applied the log-rank test to determine if the overall attrition pattern differed by gender within the colorectal cancer cohort (the only cohort that included both men and women) and found that the dropout pattern differed significantly (*P*=.02). The Kaplan-Meier curves show that females tended to have higher attrition rates than males (Figure 5), especially after Question 5.

We performed a Cox proportional hazards regression to examine whether the relationship between gender and dropout was confounded by demographic and other patient characteristics. Bivariate analyses of ethnicity, race, preferred language, recruitment phase, insurance type, and age, when compared with time to dropout, suggested that recruitment phase was the only covariate associated with survey completion (*P*=.03). After checking the proportional hazards assumption, gender, recruitment phase, and their interaction were entered into a multivariate model. The interaction was not significant, thus recruitment phase was determined to not be an effect modifier (*P*=.98). In the final model, which was adjusted for recruitment phase, it was found that gender was not significantly associated with time to dropout (*P*=.07), suggesting that attrition patterns did not differ by gender when adjusting for the recruitment phase.

## Discussion

### Principal Findings

Using our test case, visualization allowed us to identify the two most obvious points of attrition, Questions 4 and 6, with the overall attrition rate converging to approximately 60%. The use of the GLMM helped confirm these as points of significant attrition and chi-square analyses suggest that participant responses from prior questions were associated with dropping out at these points. Overall, survival analyses suggested that IDM module dropout was significantly associated with gender, implying that survey content was biased toward men, but not after accounting for recruitment phase. Furthermore, survey completion was positively associated with getting the cancer screening test. Despite the yearlong effort to create the IDM module, including focus groups, question testing, and several revisions [18], our use of the proposed framework show that this new instrument can be improved.

The proposed framework suggests that we plot overall attrition to identify patterns, analyze these patterns for significance, and then investigate potential reasons for dropout throughout the module. As the first step in evaluating attrition, visualization provides a broad view of dropout patterns throughout a survey, such as visual approximations of Eysenbach’s curiosity, attrition, and stable use phases [4]. Even if questions that appear to have high dropout in this step do not turn out to be statistically significant points of attrition, this step still highlights questions that might be too complex, poorly worded, or provide enough information that participants do not feel the need to continue further.

Prior work in this area has encountered challenges. For example, Ekman plotted the number of dropouts per question on 2 surveys in a bar chart, revealing that most of the dropout occurred within the first 8 questions [1]. Although the grouped bar chart was informative in this instance, this type of plot has the limitation of appearing crowded and is difficult to interpret if it includes several groups. Ekman also employed the use of step functions, although high response rates made it difficult to identify questions with high attrition [1]. Whereas the survival-type curve can be a useful visualization tool, it may be less informative when attrition is low. Hoerger used a step function to compare attrition between 6 surveys, but inconsistent survey lengths made it difficult to compare the attrition rates [15]. An advantage of step functions and survival-type curves is that they can display skip patterns, whereas the survival analysis setting does not allow researchers to take this into account.

The second stage of our dropout attrition framework is designed to confirm whether certain drops in response rates are significant. These formal statistical analyses can not only confirm observed trends from the visualizations, but also locate differences that were not observable.

The last stage proposes an examination of possible causes of participant dropout. Collecting and adjusting for demographic characteristics (especially those previously suggested as predictive of survey completion including gender, age, education, and ethnicity) [13,20] may identify biases in the survey content or wording of survey items. The association between participant responses and dropout in the next question may suggest which patients are most interested in the survey or what content retains more respondents. Prior research suggests that relevant survey content is actually more predictive of dropout attrition than overall survey length [13-15]. This framework allows researchers to identify “problem questions” and adjust content when appropriate.

### Limitations

As noted in the Introduction, the methods proposed here are meant only as a starting point. These methods could additionally be considered as a part of the survey testing process in helping to refine the instrument and retain the maximum number of participants. This paper does not discuss other forms of attrition that apply to online surveys, such as nonresponse attrition, attrition in longitudinal surveys, or methods to minimize attrition or correct for potential bias introduced by high attrition rates.

### Future Work

Although not exemplified in this paper, discrete time survival analysis would be a more appropriate though more complex method to identify this type of survival pattern as patients can only drop out at discrete time points (ie, after each question). We applied the GLMM pairwise to our case study but it is also possible to fit a single model to the entire survey, though this complex modeling would require more sophisticated parameterization (eg, dependence structures) that may affect estimator accuracy and convergence. The indicator used in our GLMM distinguished whether a patient answered a survey question or not, but could have instead indicated whether the respondent dropped out at a particular question. Results will not be the exact inverse in cases where respondents are allowed to skip questions.

These analyses can be enhanced by linking responses to subject characteristics or metadata. Online surveys provide additional information not previously available in paper-based surveys, perhaps most notably metadata. The amount of time a patient spends on each question, the time of day a survey is taken, and Internet browser version compatibility are all examples of metadata that could also affect attrition patterns.

Survey characteristics associated with overall completion, such as survey relevance, could also be examined question by question [13]. In addition, although we suggest several types of factors that may be associated with attrition (and analyzed them separately in our test case), we acknowledge that it may also be useful to look at these factors simultaneously. It is up to the discretion of the researcher to determine whether or not to look at these factors separately or together in a model-based method, such as multiple logistic regression.

### Conclusions

We contend that simply reporting attrition rates is not enough; we must dig deeper to examine where and why attrition occurs. Our contribution here is to advocate advances in the science of attrition. The framework outlined in this manuscript is especially important when fielding new surveys that have not been previously tested or validated. This framework is best applied as both part of the survey development process and as a tool for interpreting survey results. We encourage researchers to engage with these steps throughout the research process as we work as a community to establish a “law of attrition.”

## Multimedia Appendix 1

MyQuestions Informed Decision Making Module.

## Abbreviations

EHR: electronic health records
GLMM: generalized linear mixed model
IDM: informed Decision-Making
MPC: MyPreventiveCare
RCT: randomized controlled trial
