# Supplementary material for: Methods for Evaluating Respondent Attrition in Web-Based Surveys
Source: J Med Internet Res. 2016 Nov 22;18(11):e301. doi: 10.2196/jmir.6342 (PMC5141338; doi:10.2196/jmir.6342)
Supplement: Supplementary file 1 [file jmir_v18i11e301_app1.pdf]

MyPreventiveCare patient portal home page

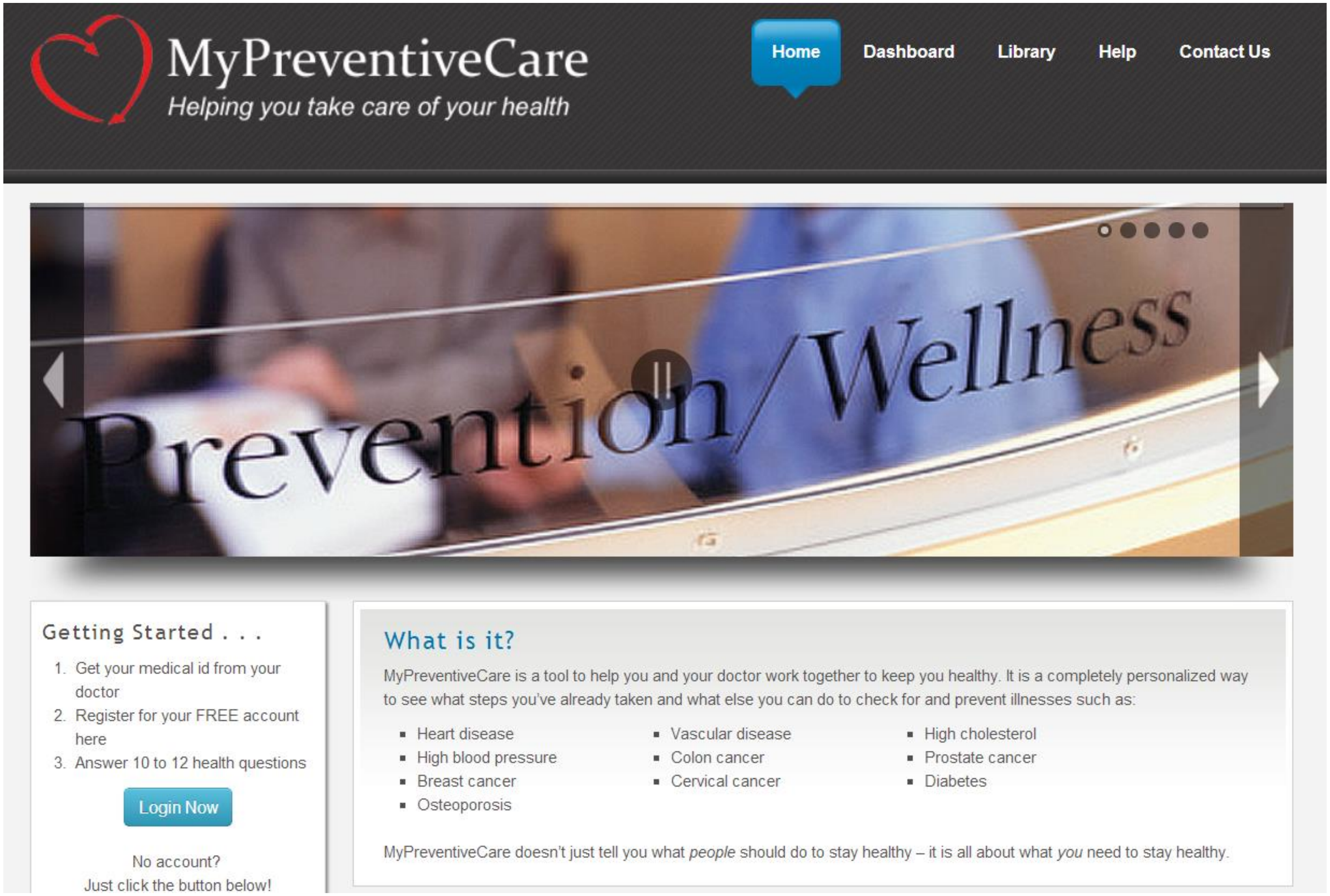

Alert to start *MyQuestions* in the patient dashboard

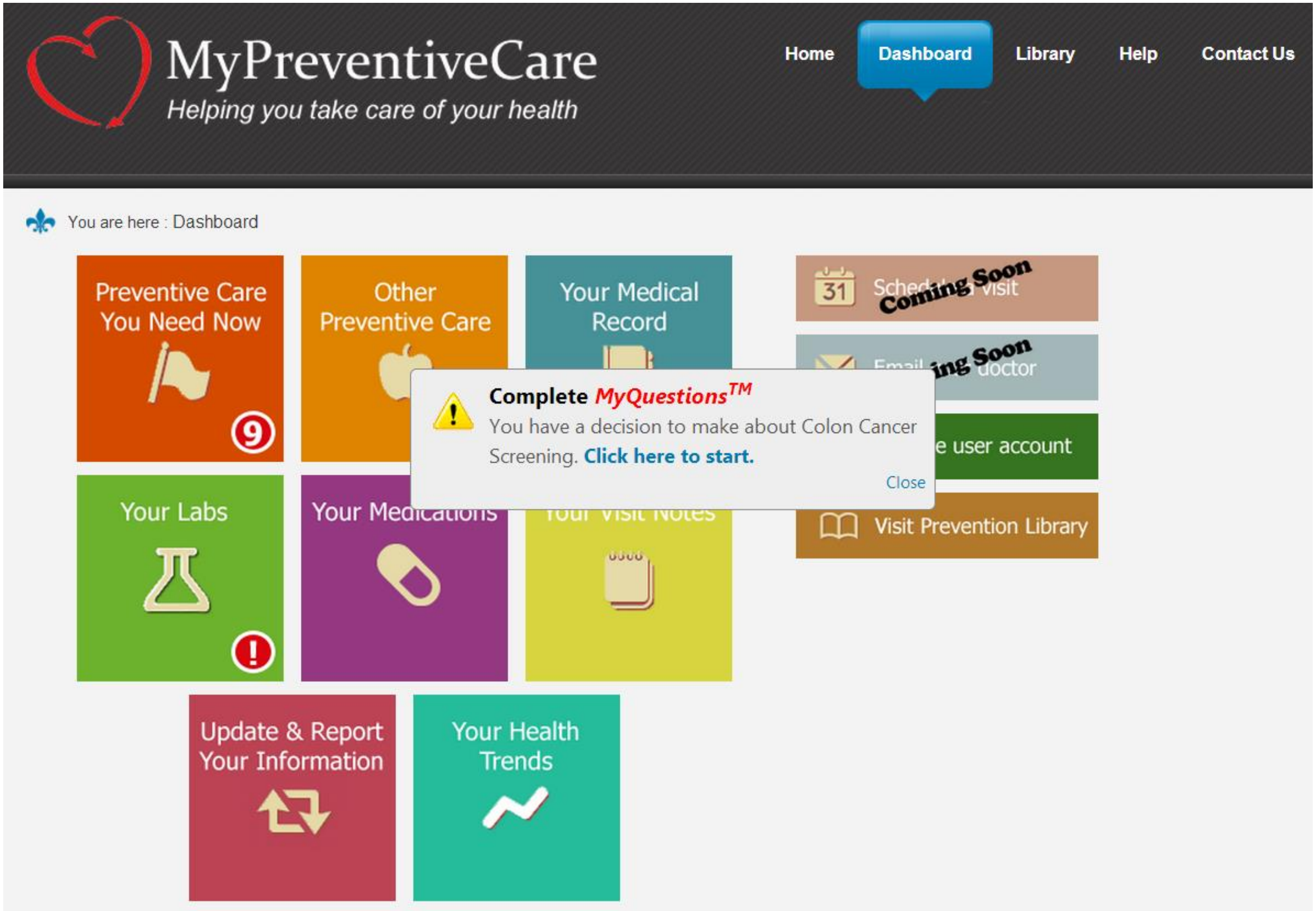

MyQuestions opening page

MyPreventiveCare

Helping you take care of your health

Home Dashboard Library Help Contact Us

You are here : MyQuestions

The Decision about Breast Cancer Screening

Your Information

You have never had a mammogram.

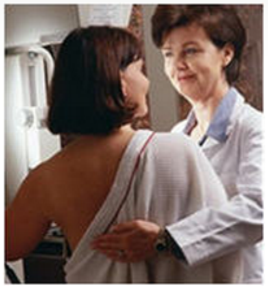

The Decision

The decision to get screening mammograms between the age of 40 and 49 years is an individual one. It depends on your risk for breast cancer. It also depends on your values about the benefits and harms.

Is screening right for you?

We want to give you more information for this decision but first we want to know what questions are at the top of your list. Please click MyQuestions™ below and take a few minutes to help us learn how we can help you.

Start MyQuestions™

No thanks, ask me about this another time.

MyQuestions Question #1

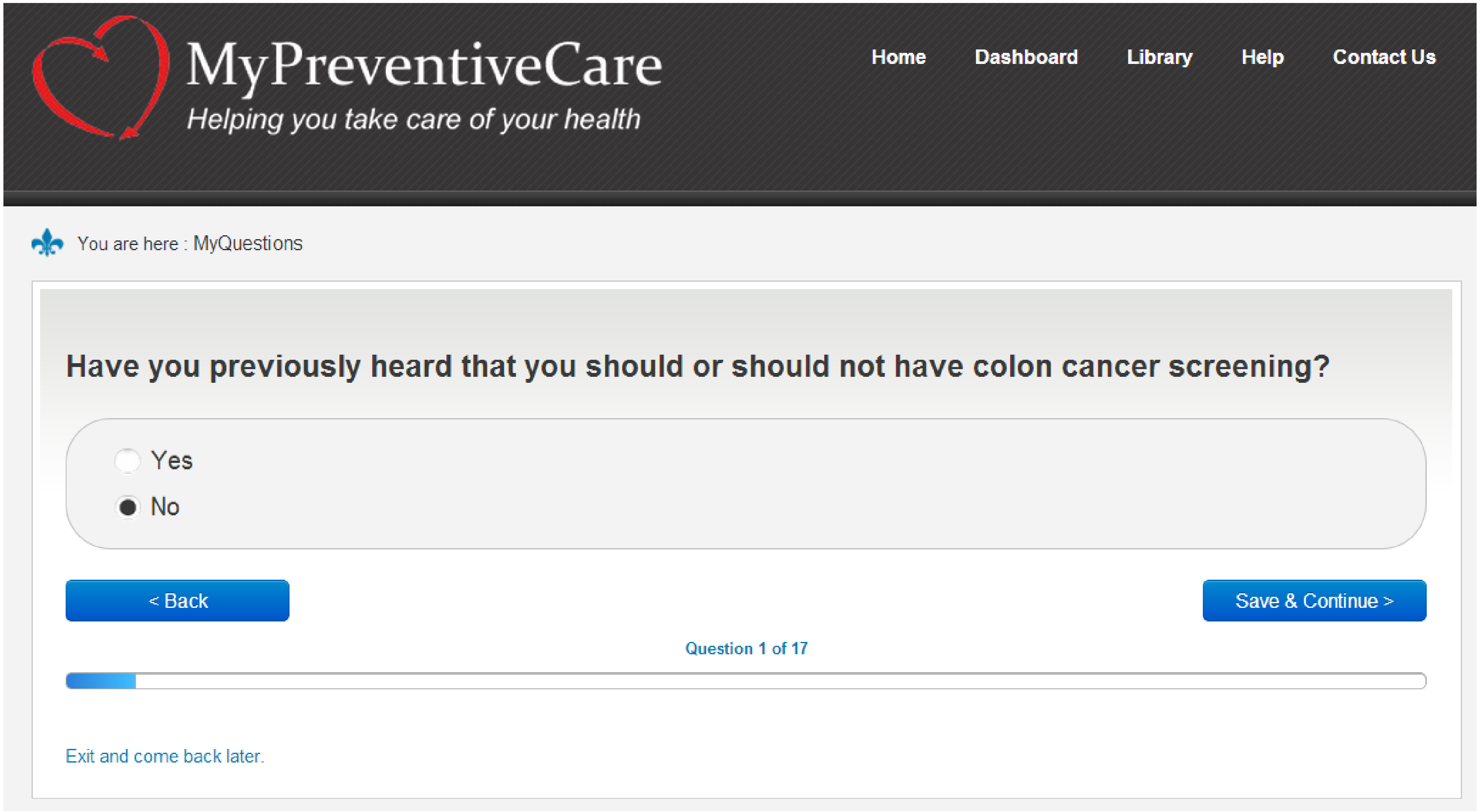

MyQuestions Question #2

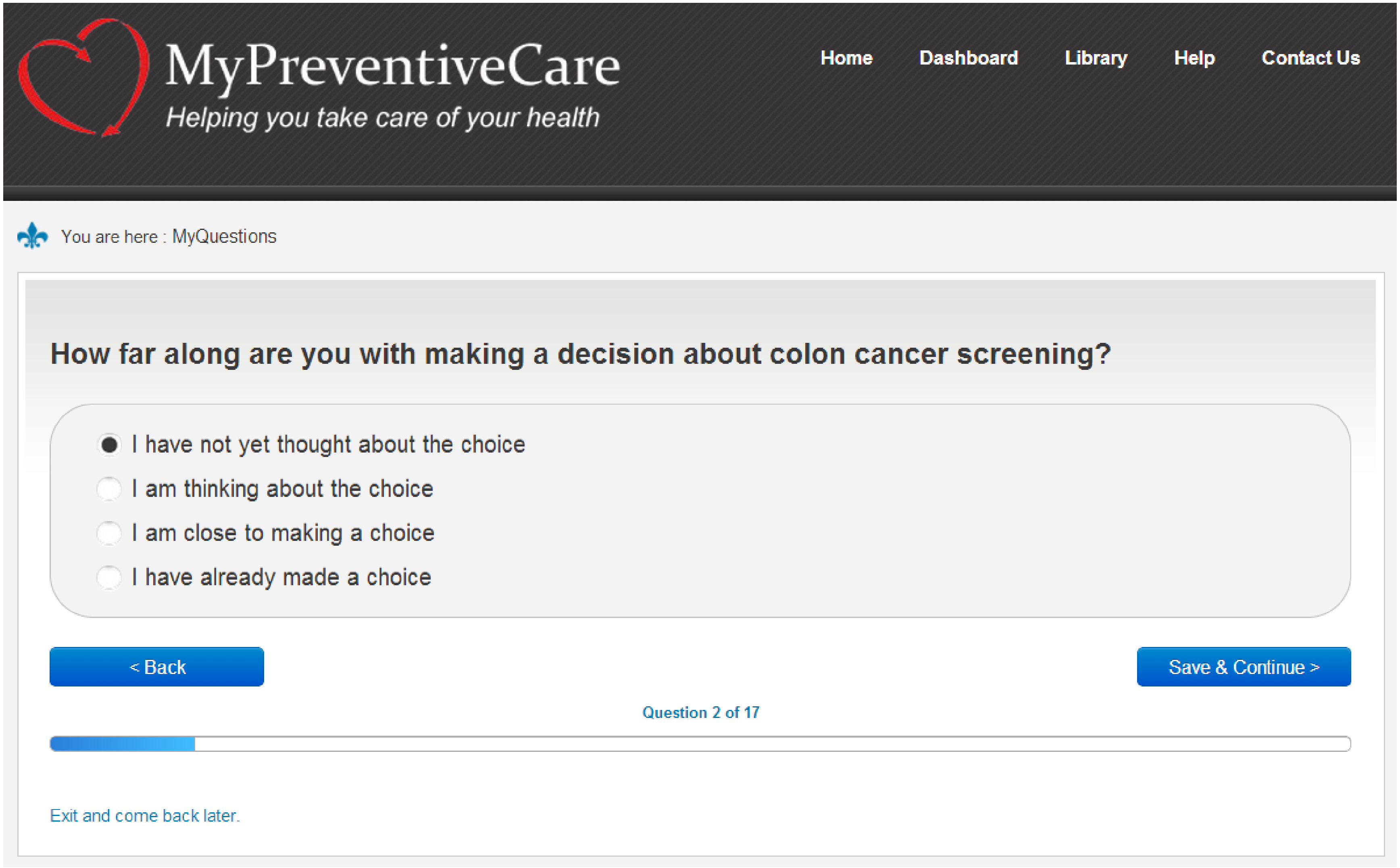

MyQuestions Question #3

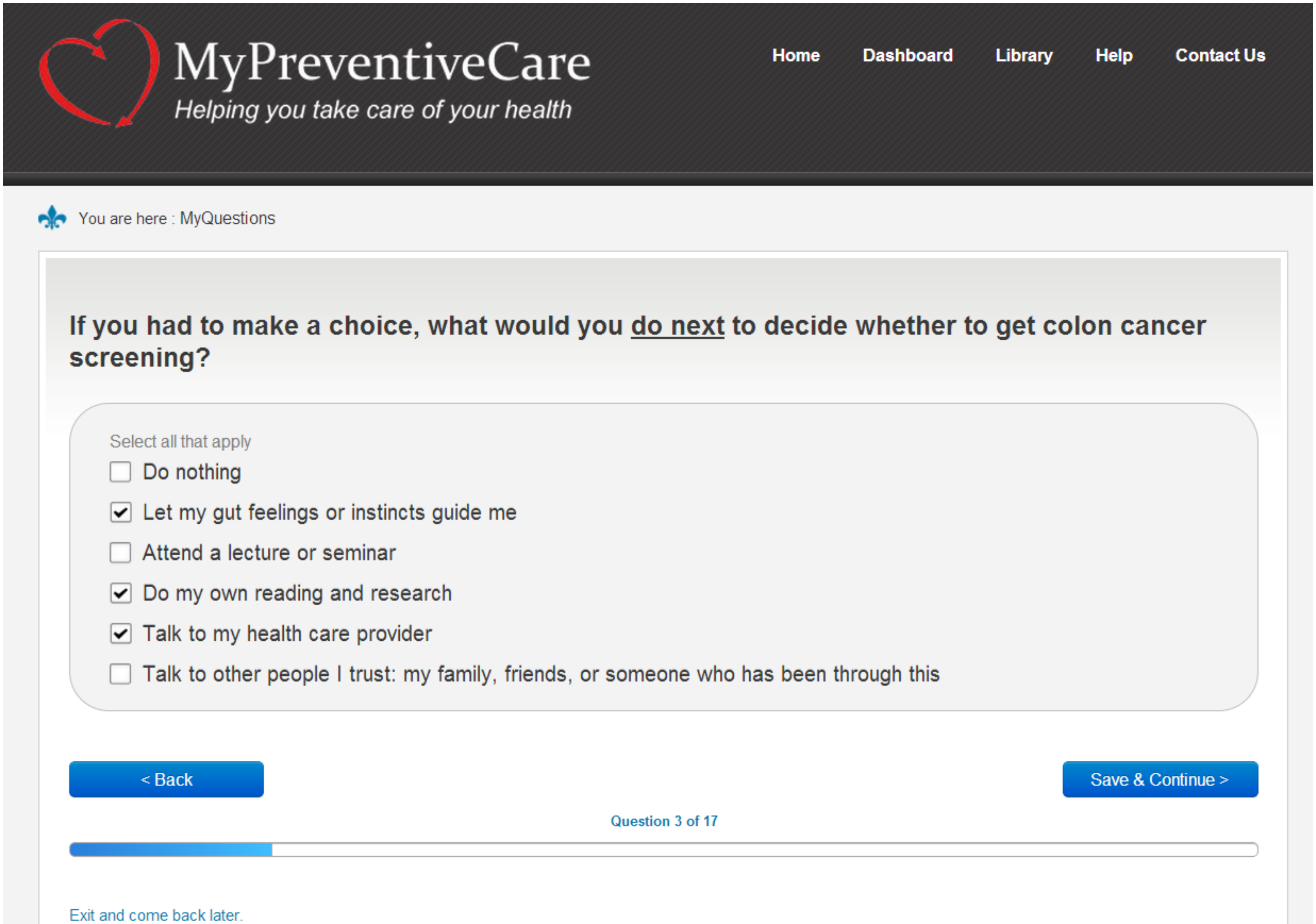

MyPreventiveCare
Helping you take care of your health

Home | Dashboard | Library | Help | Contact Us

You are here : MyQuestions

If you had to make a choice, what would you do next to decide whether to get colon cancer screening?

Select all that apply

- [ ] Do nothing
- [x] Let my gut feelings or instincts guide me
- [ ] Attend a lecture or seminar
- [x] Do my own reading and research
- [x] Talk to my health care provider
- [ ] Talk to other people I trust: my family, friends, or someone who has been through this

< Back | Save & Continue >

Question 3 of 17

Exit and come back later.

MyQuestions transition page

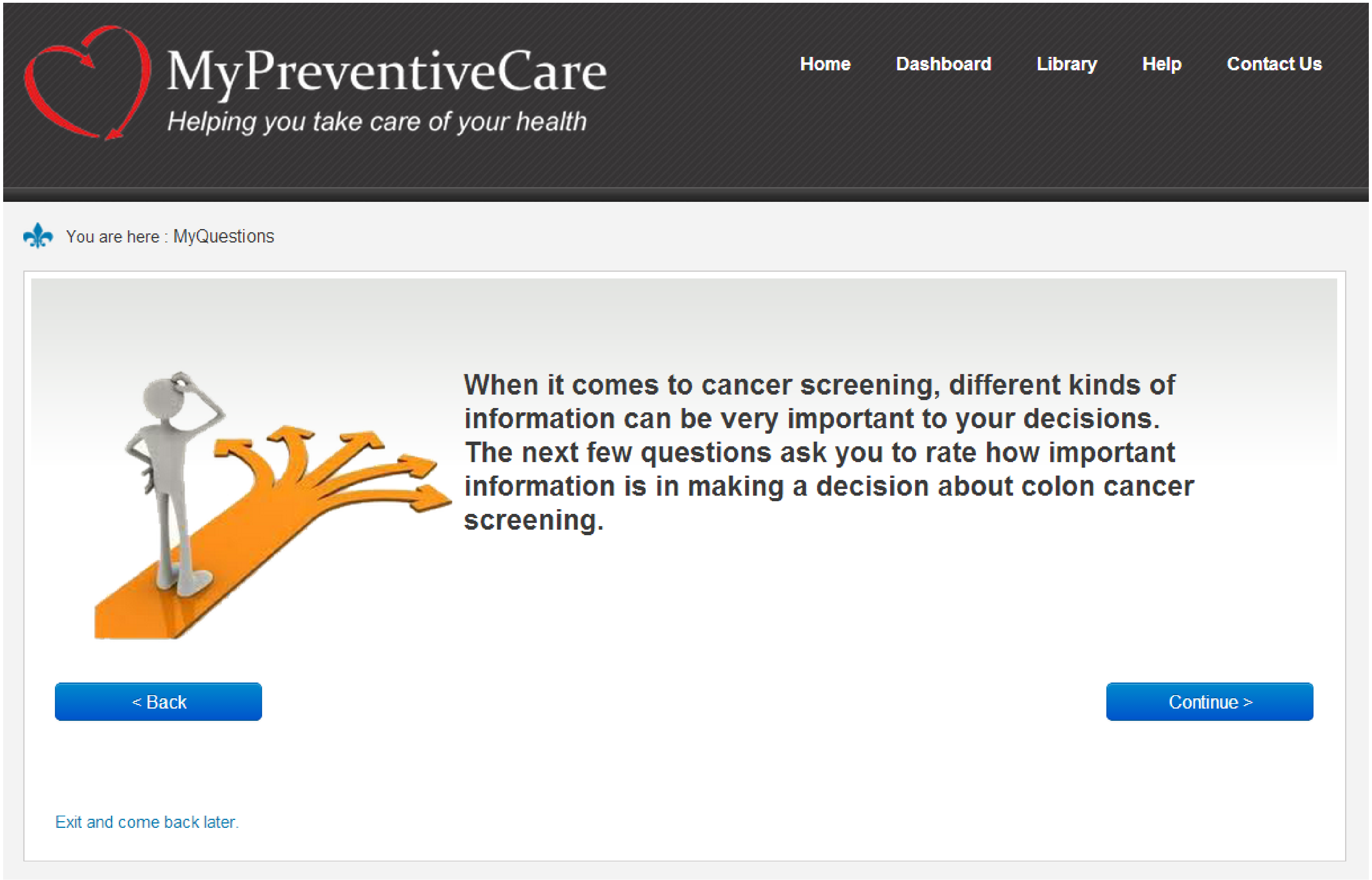

MyQuestions Question #4

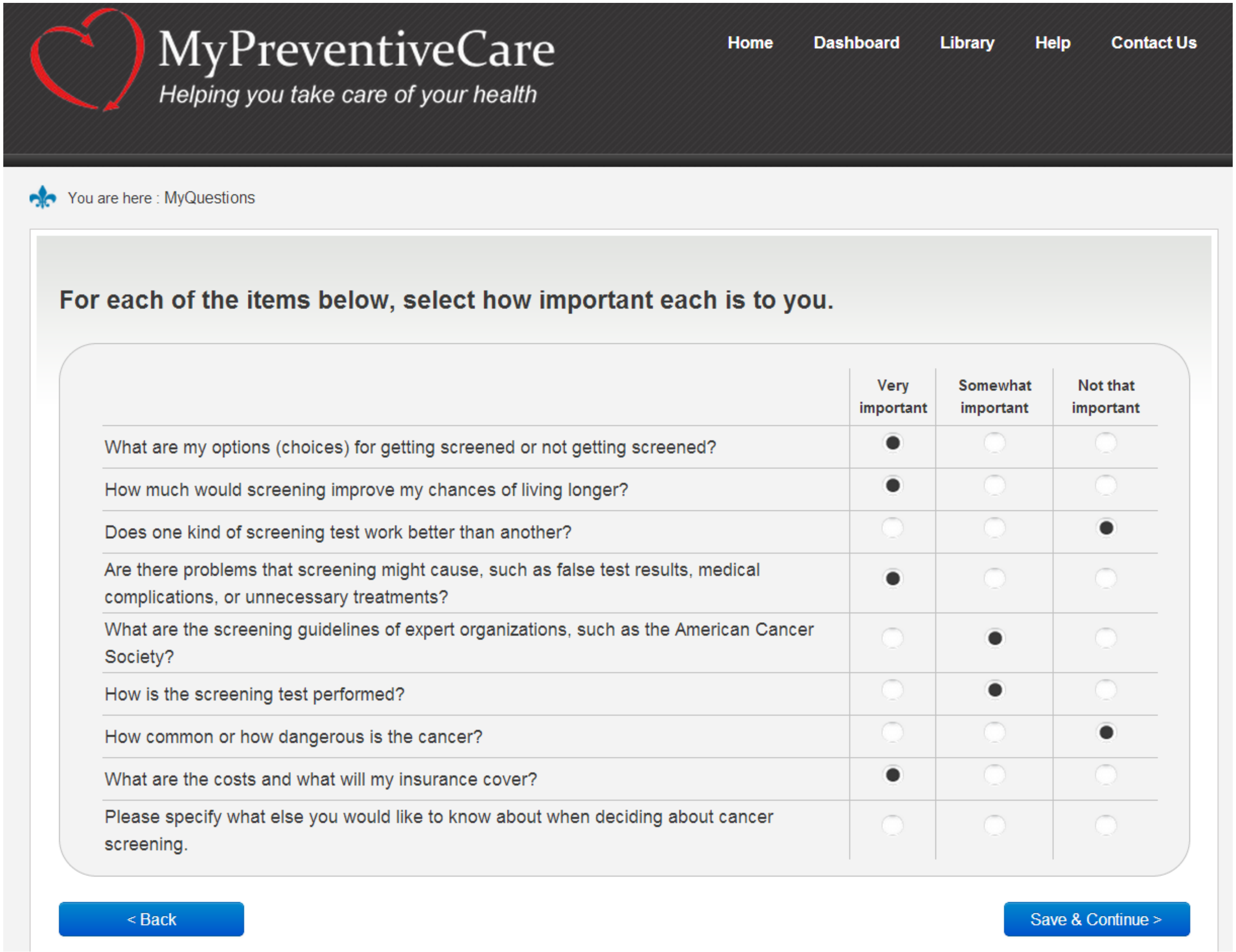

MyPreventiveCare
Helping you take care of your health

Home | Dashboard | Library | Help | Contact Us

You are here : MyQuestions

For each of the items below, select how important each is to you.

	Very important	Somewhat important	Not that important
What are my options (choices) for getting screened or not getting screened?	●	○	○
How much would screening improve my chances of living longer?	●	○	○
Does one kind of screening test work better than another?	○	○	●
Are there problems that screening might cause, such as false test results, medical complications, or unnecessary treatments?	●	○	○
What are the screening guidelines of expert organizations, such as the American Cancer Society?	○	●	○
How is the screening test performed?	○	●	○
How common or how dangerous is the cancer?	○	○	●
What are the costs and what will my insurance cover?	●	○	○
Please specify what else you would like to know about when deciding about cancer screening.	○	○	○

< Back | Save & Continue >

MyQuestions transition page

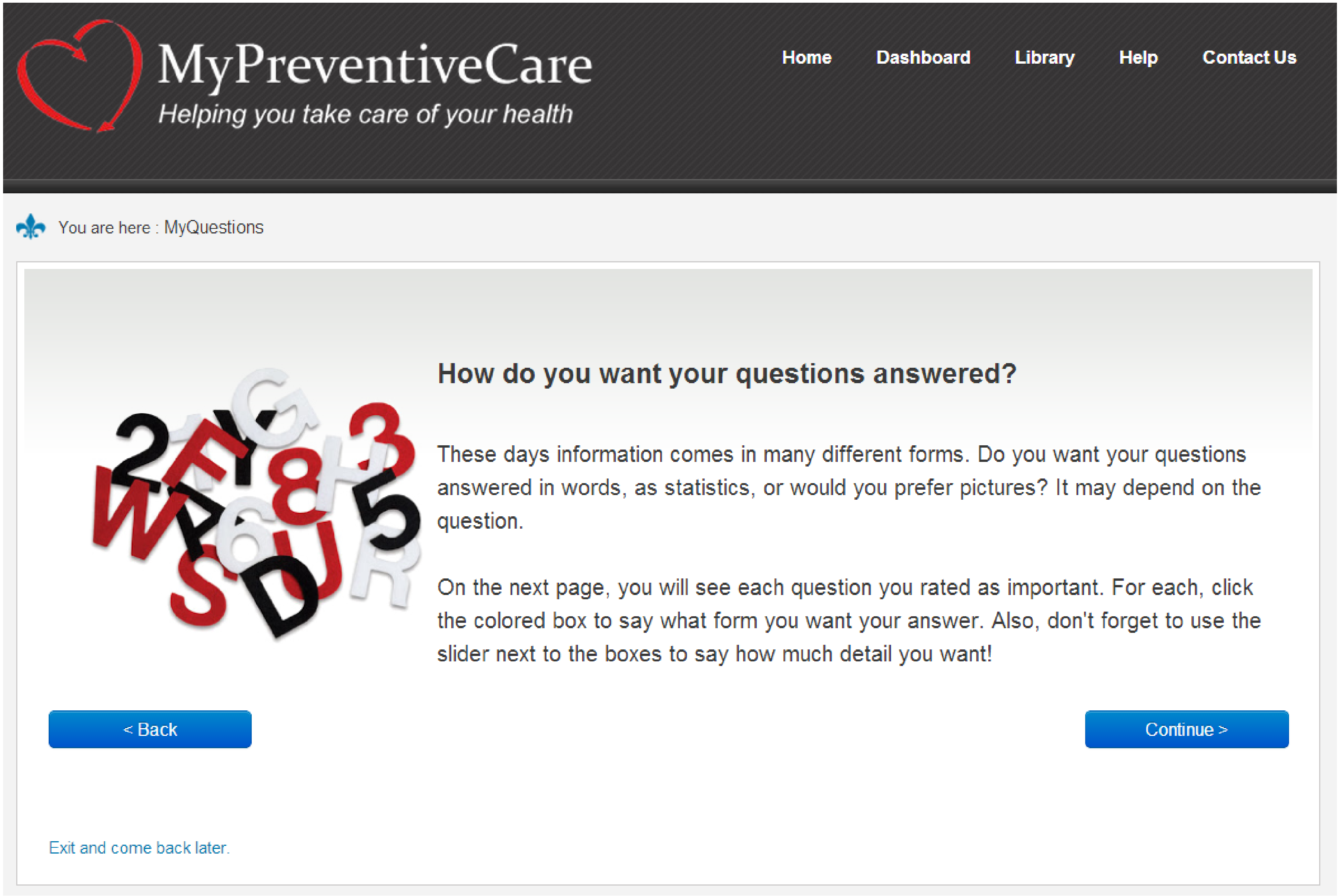

MyQuestions Question #5

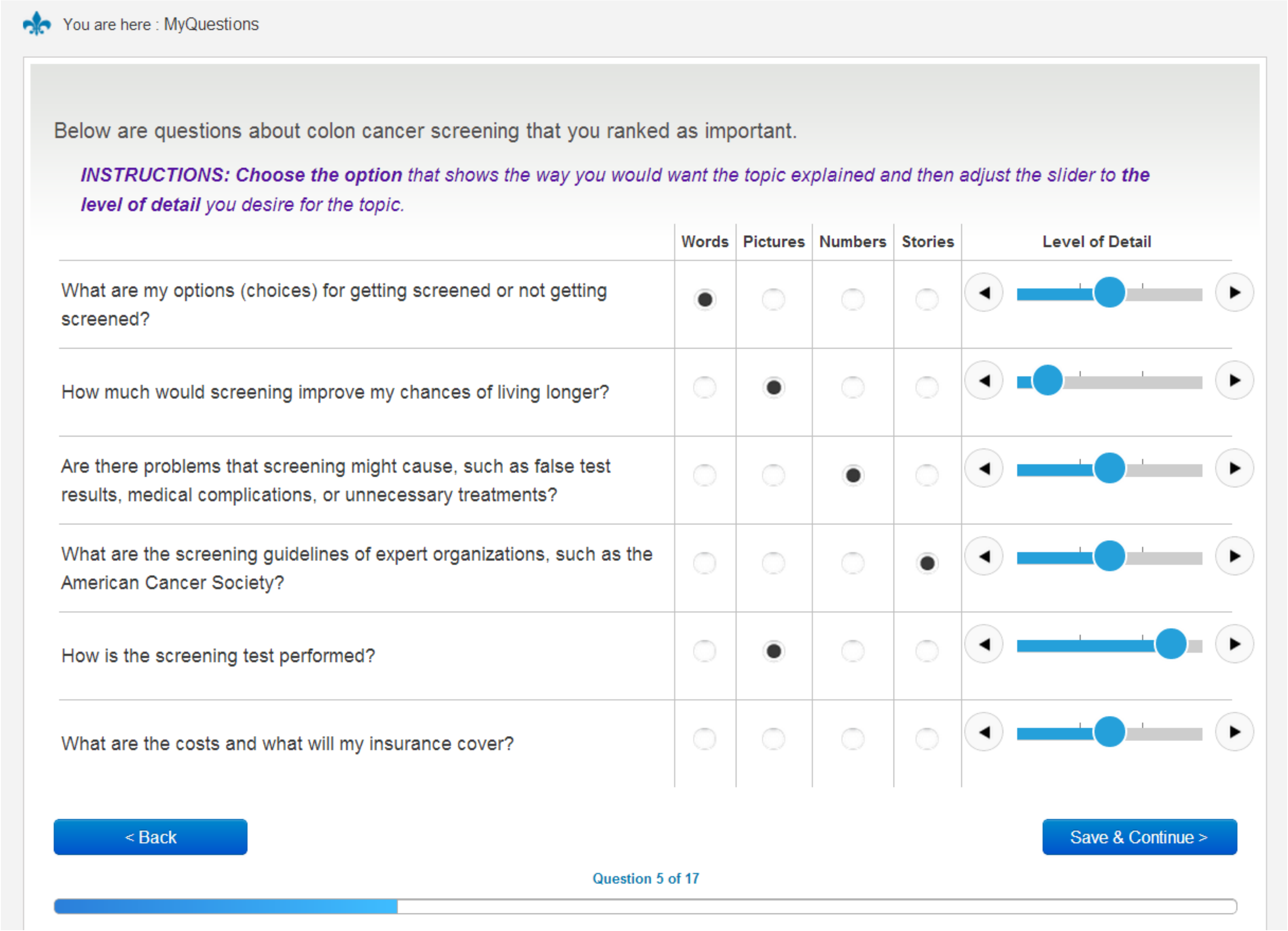

MyQuestions Question #6

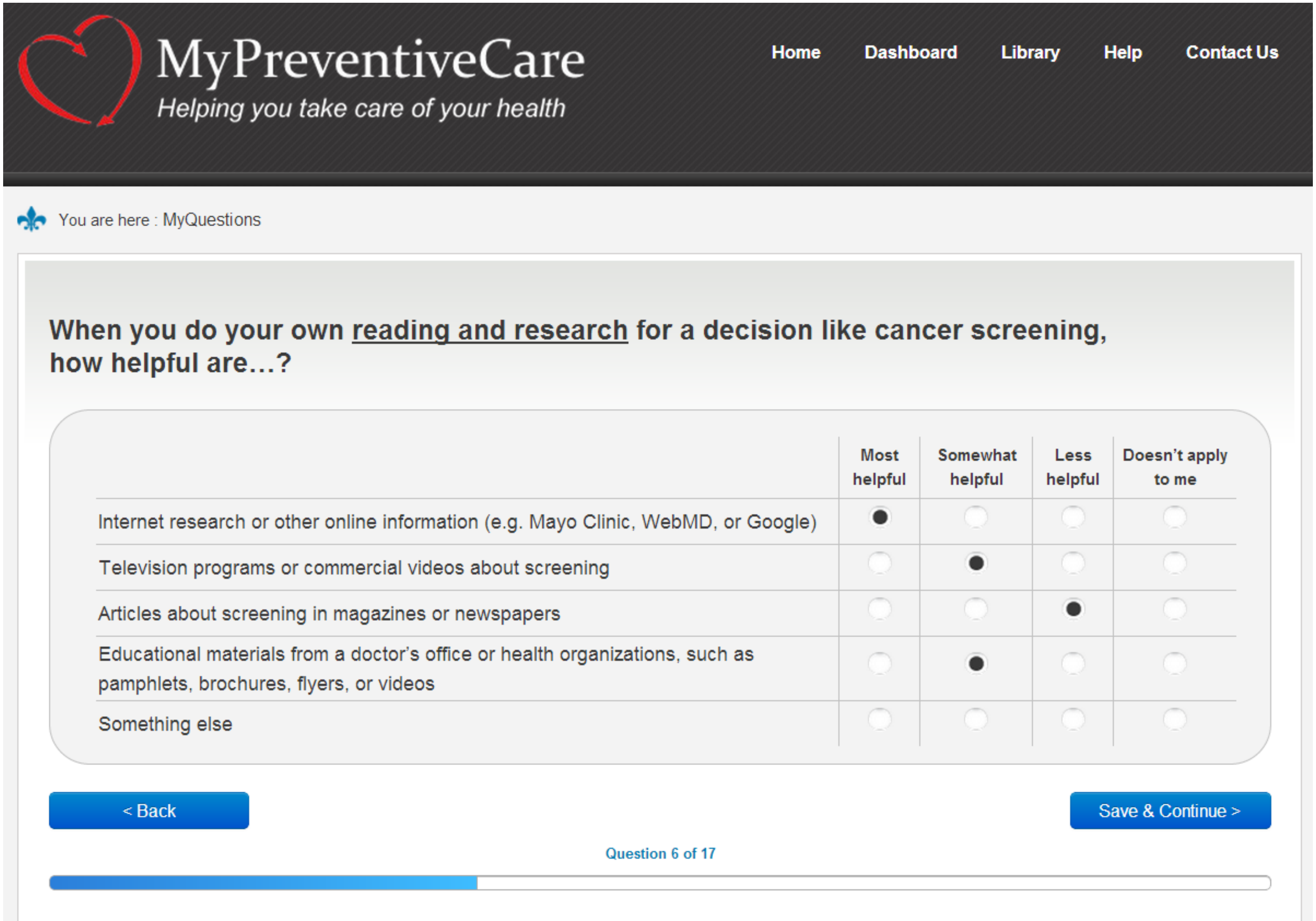

MyQuestions Question #7

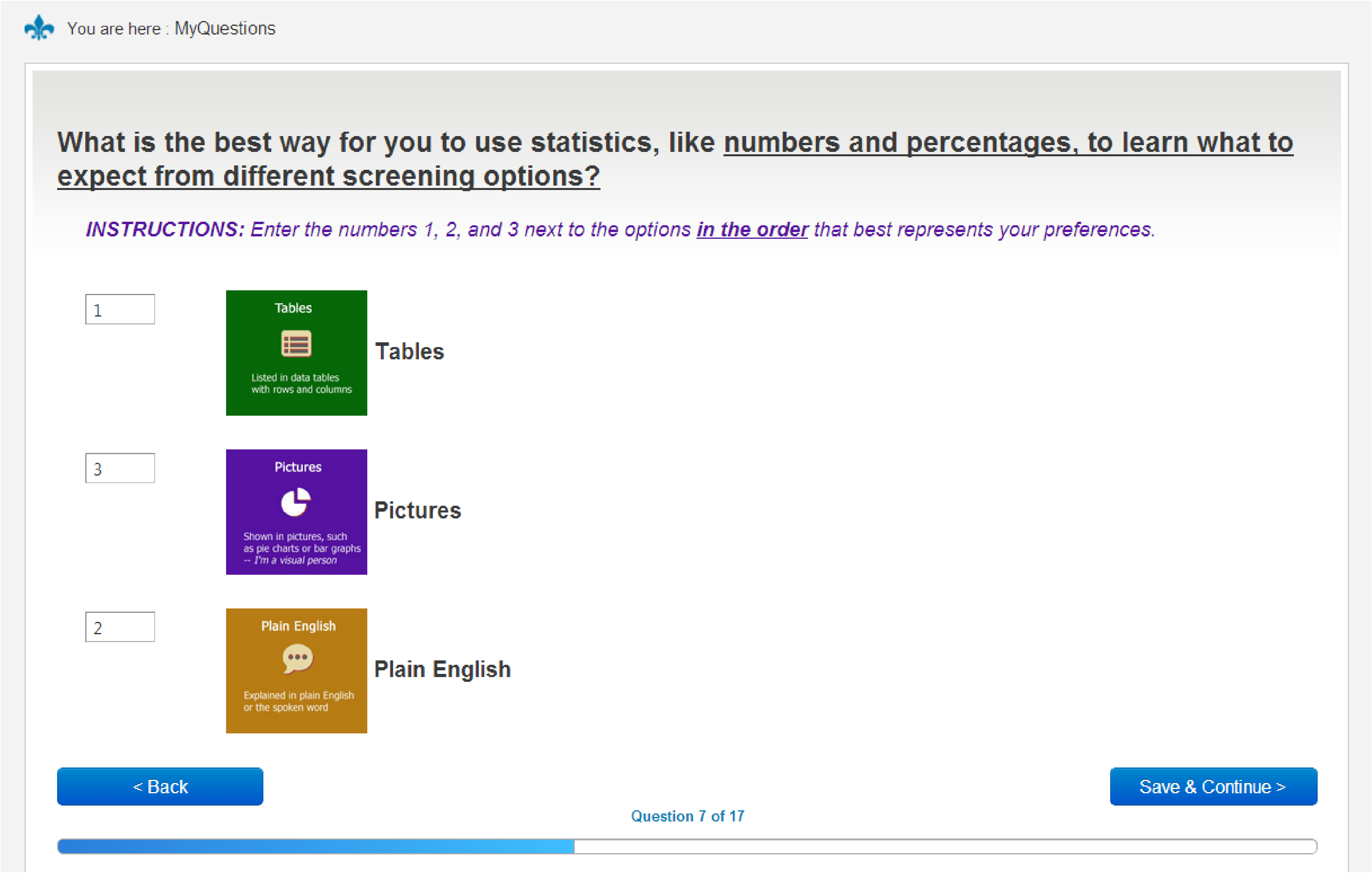

MyQuestions transition page

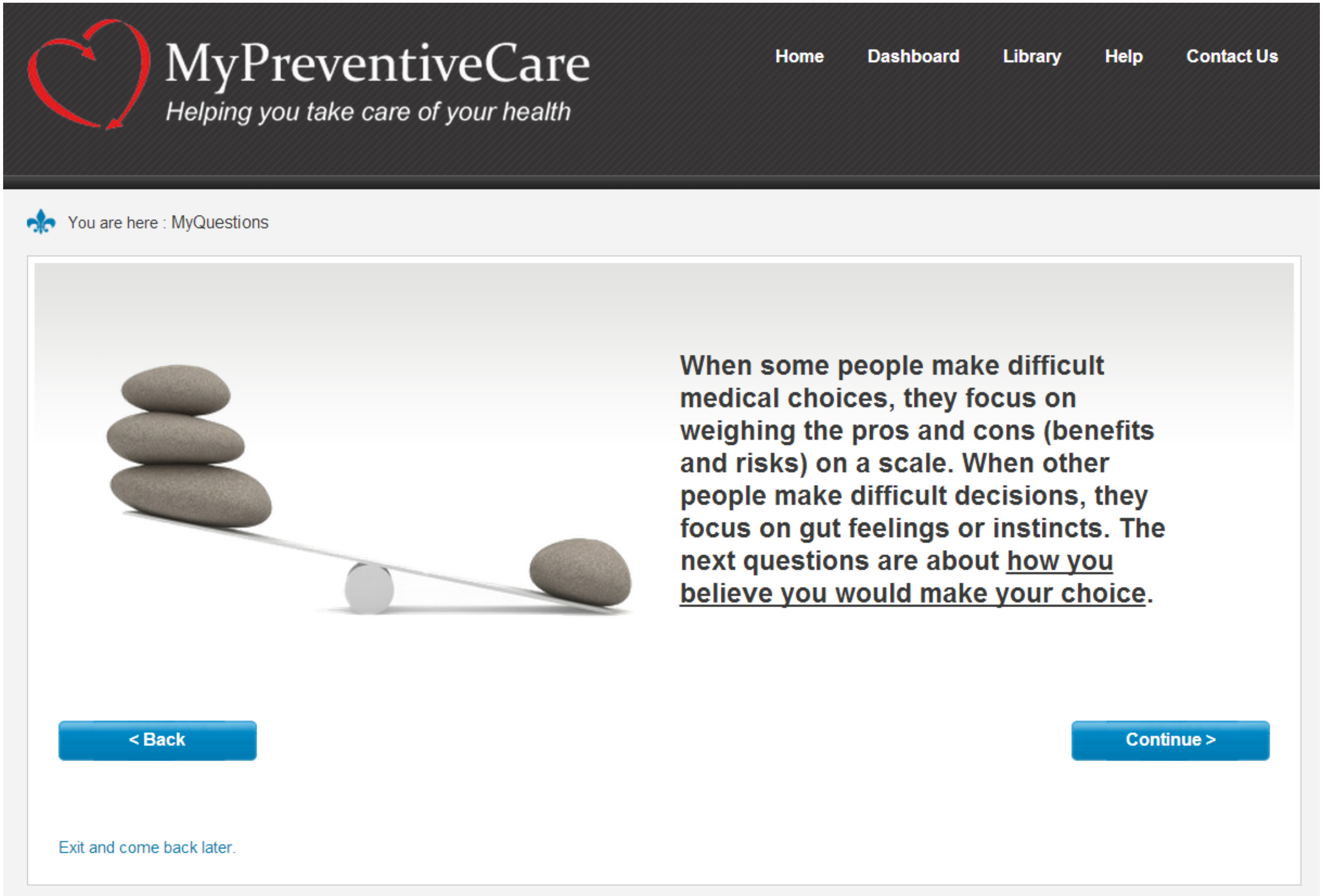

MyQuestions Question #8

You are here : MyQuestions

How important to you are your gut feelings or instincts about cancer screening compared with weighing the pros and cons?

***INSTRUCTIONS:** Drag the slider below to the response that represents how you believe you would make your choice.*

▲

Gut feelings or instincts are most important

Gut feelings or instincts are somewhat important

Not sure or neutral

Weighing pros and cons are somewhat important

Weighing pros and cons are most important

▼

< Back

Save & Continue >

Question 8 of 17

Exit and come back later.

MyQuestions Question #9

You are here : MyQuestions

How important are the following fears or worries to your decision about colon cancer screening?

	Very important	Somewhat important	Not that important
Getting cancer or not catching cancer early enough.	●	○	○
The pain or embarrassment of the test.	○	○	●
Getting bad news when the results come back or finding out I have cancer.	○	●	○
The costs of screening.	○	●	○
Having complications (risks and side effects) from the screening test.	●	○	○
Going to health care providers.	○	○	●
Regretting my decision.	○	○	●
Please specify what else you would like to know about when deciding about cancer screening.	○	○	○

< Back

Save & Continue >

Question 9 of 17

Exit and come back later.

Patient created “decision aid”
Tiles with information patient said was “very important” (question 4) in the format they wanted to see the information (question 5). Up to four tiles were included, although patients could access all resources (for all topics and all formats) by clicking on “More information about Colon Cancer Screening.”

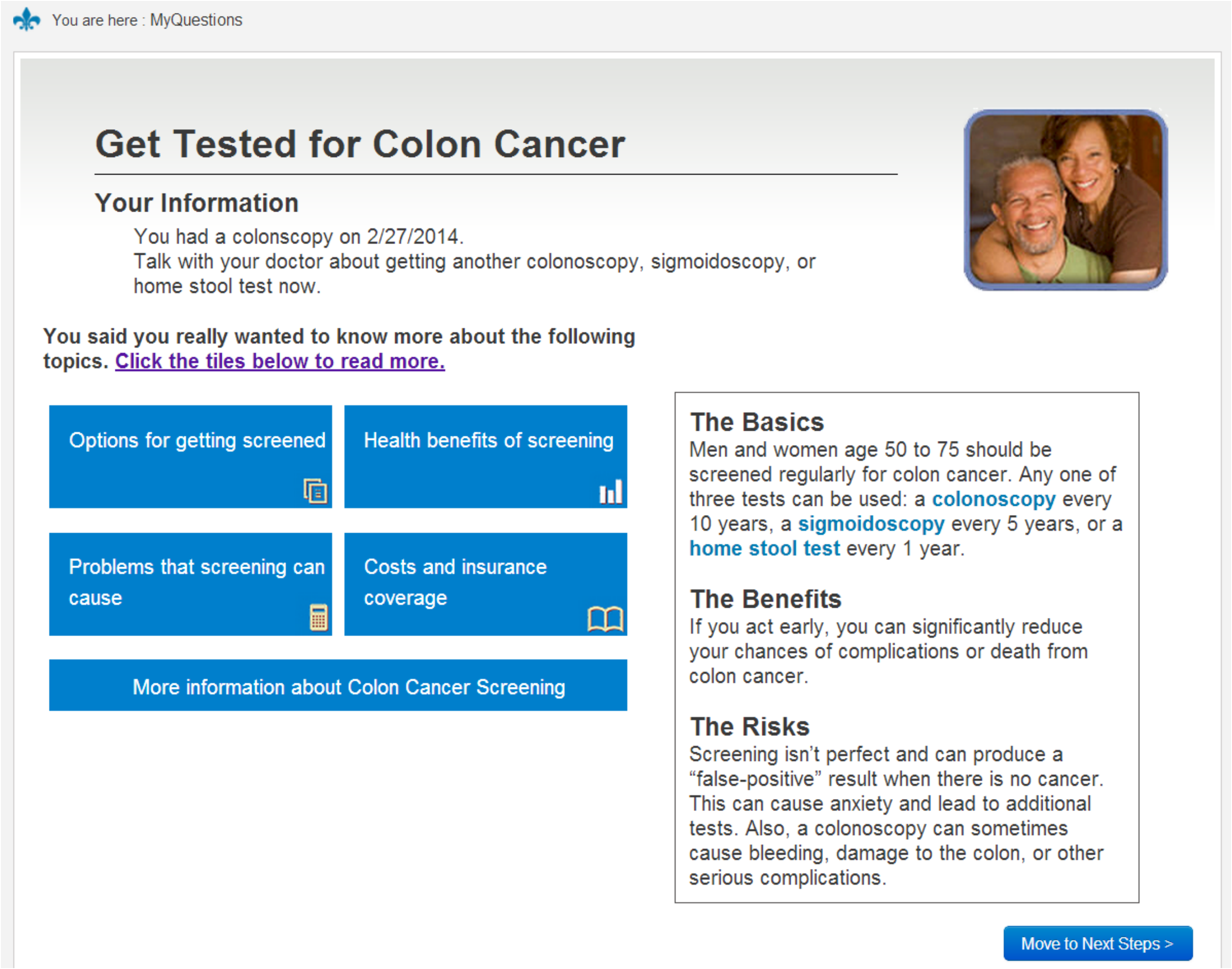

Example of content in patient educational material tile

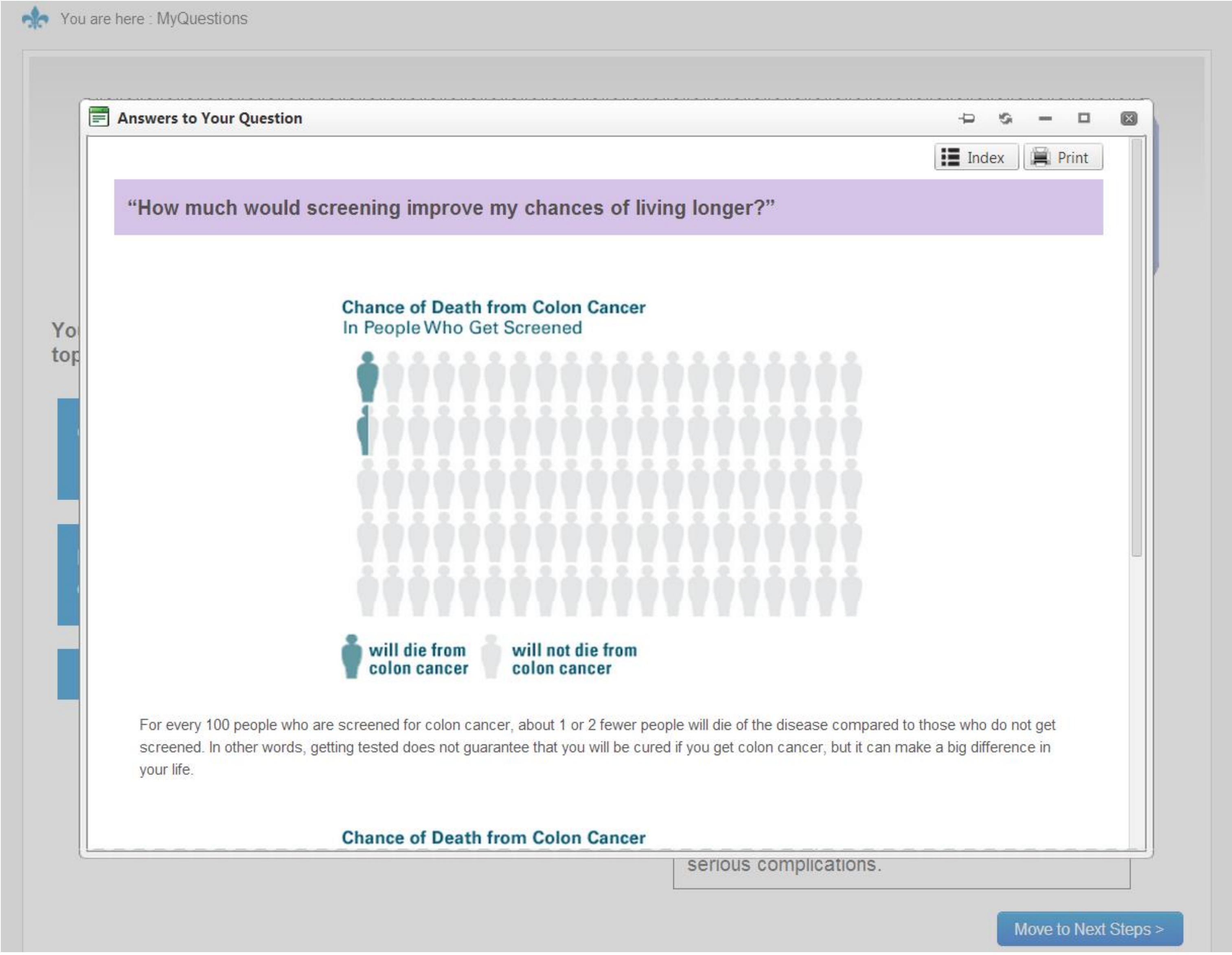

Patient can access a library of all educational material

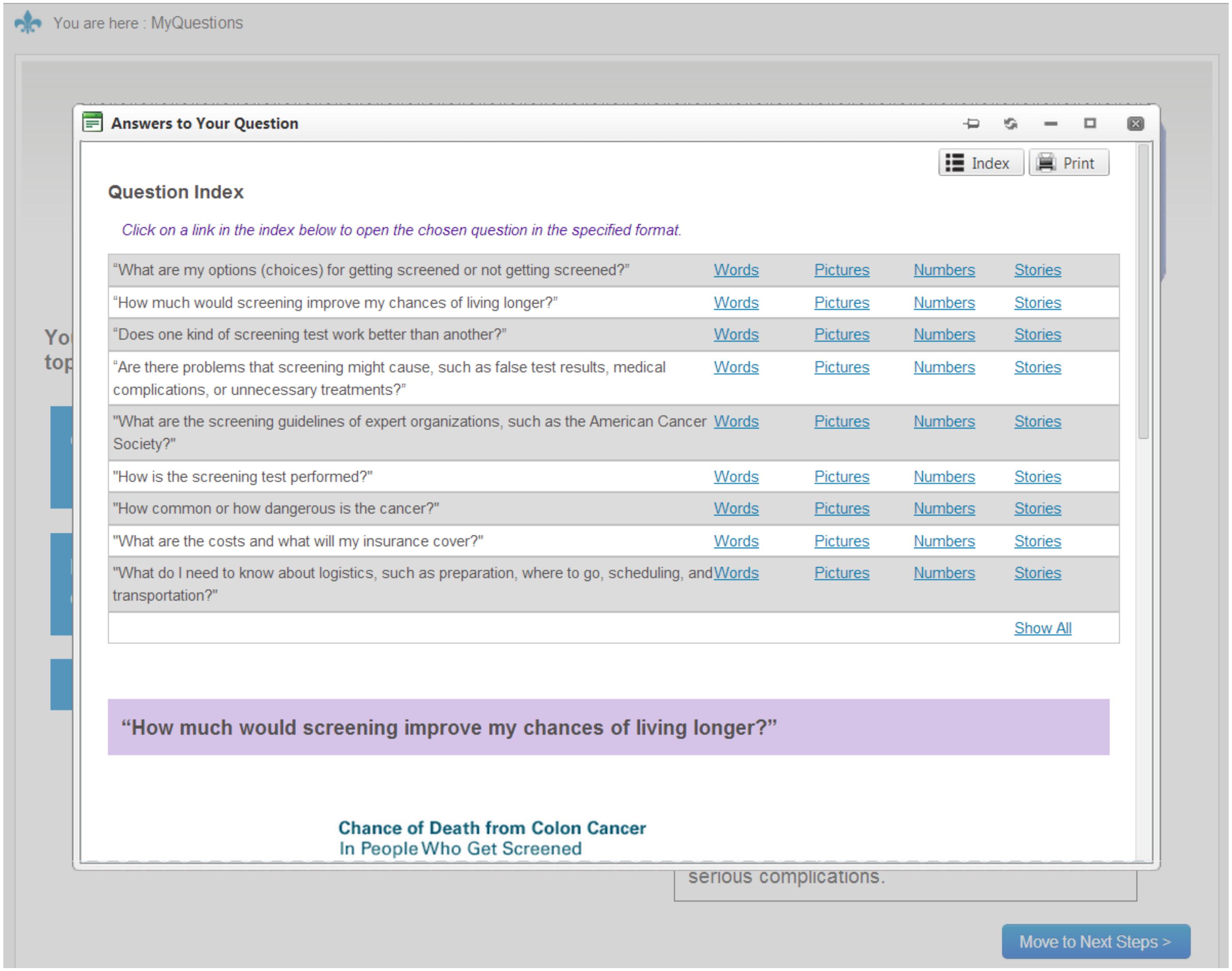

MyQuestions Question #10

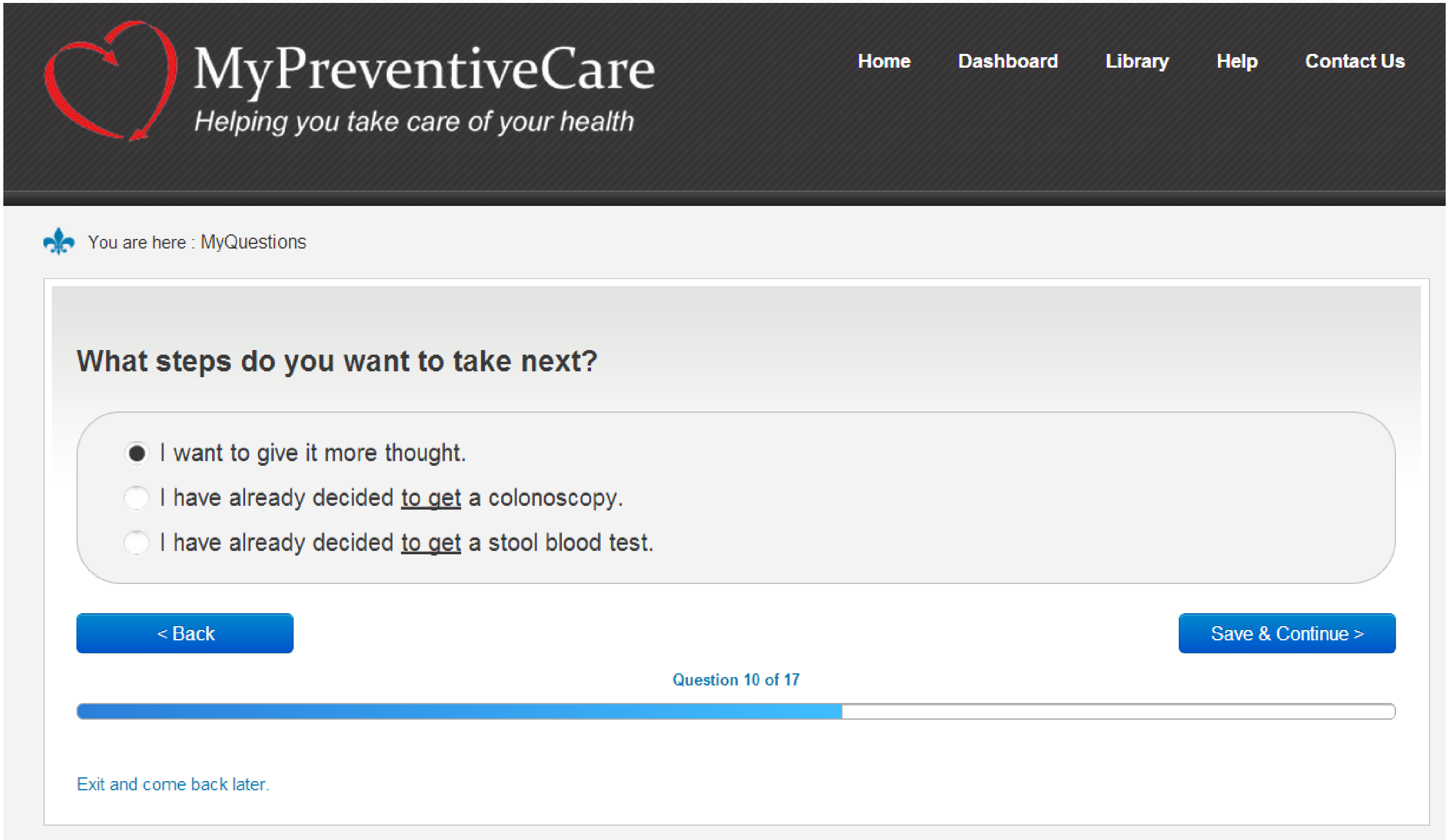

MyQuestions Question #11

MyPreventiveCare
Helping you take care of your health

Home | Dashboard | Library | Help | Contact Us

You are here : MyQuestions

Which of the following would you like to do?

- [ ] No next steps for now.
- [ ] Do more reading and research.
- [ ] Fill out a printed or online “decision aid” questionnaire that could help me weigh pros and cons.

Talk with . . . *[check all that apply]*

- [x] . . . my regular health care provider.
- [ ] . . . a specialist for a second opinion.
- [x] . . . my family, friends, clergy, or other people I trust.

- [ ] Prayer.
- [x] Get a reminder to make this decision.
- [ ] Other next steps.

< Back | Save & Continue >

Question 11 of 17

MyQuestions Question #12

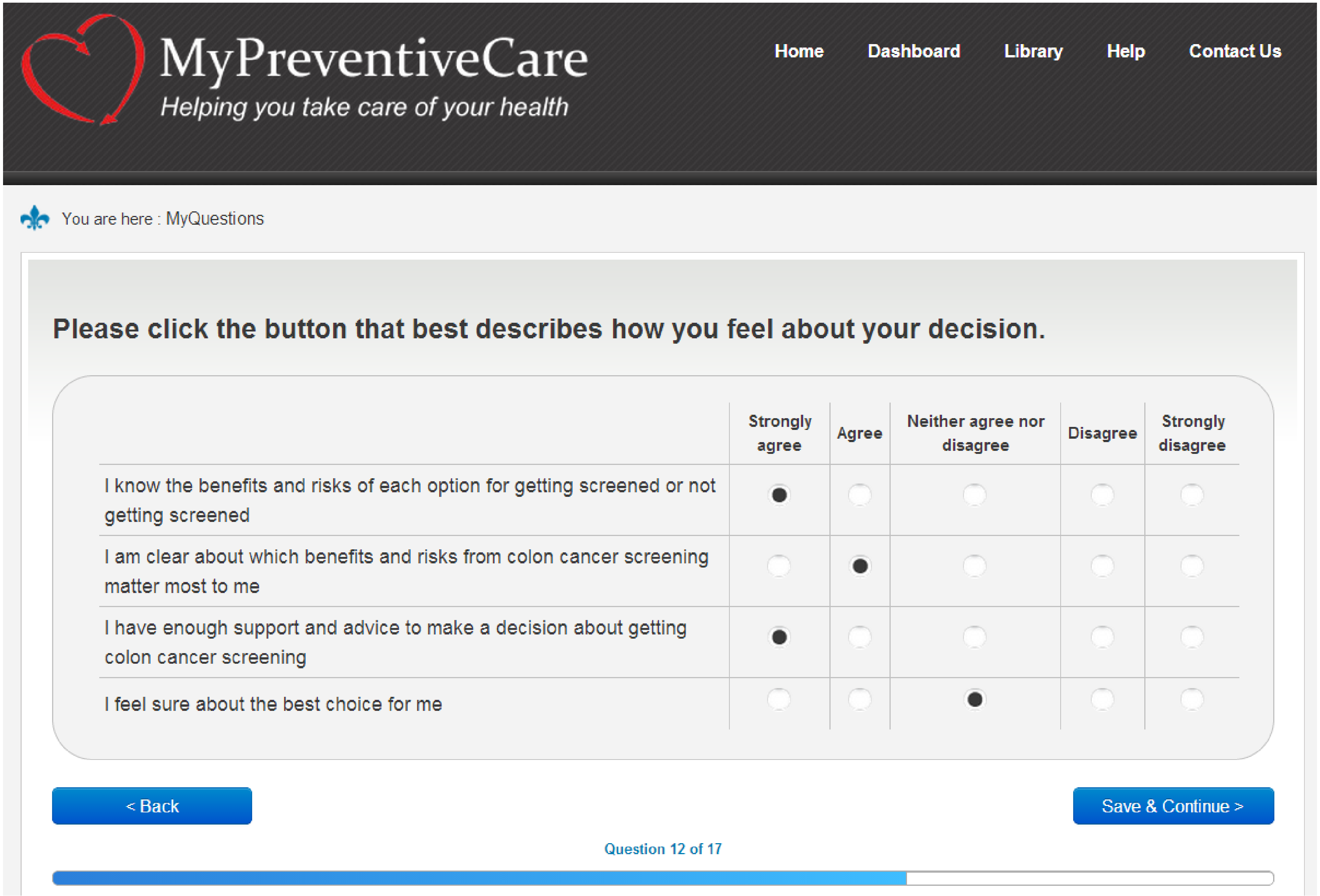

MyPreventiveCare
Helping you take care of your health

Home Dashboard Library Help Contact Us

You are here : MyQuestions

Please click the button that best describes how you feel about your decision.

	Strongly agree	Agree	Neither agree nor disagree	Disagree	Strongly disagree
I know the benefits and risks of each option for getting screened or not getting screened	●	○	○	○	○
I am clear about which benefits and risks from colon cancer screening matter most to me	○	●	○	○	○
I have enough support and advice to make a decision about getting colon cancer screening	●	○	○	○	○
I feel sure about the best choice for me	○	○	●	○	○

< Back

Save & Continue >

Question 12 of 17

MyQuestions Question #13

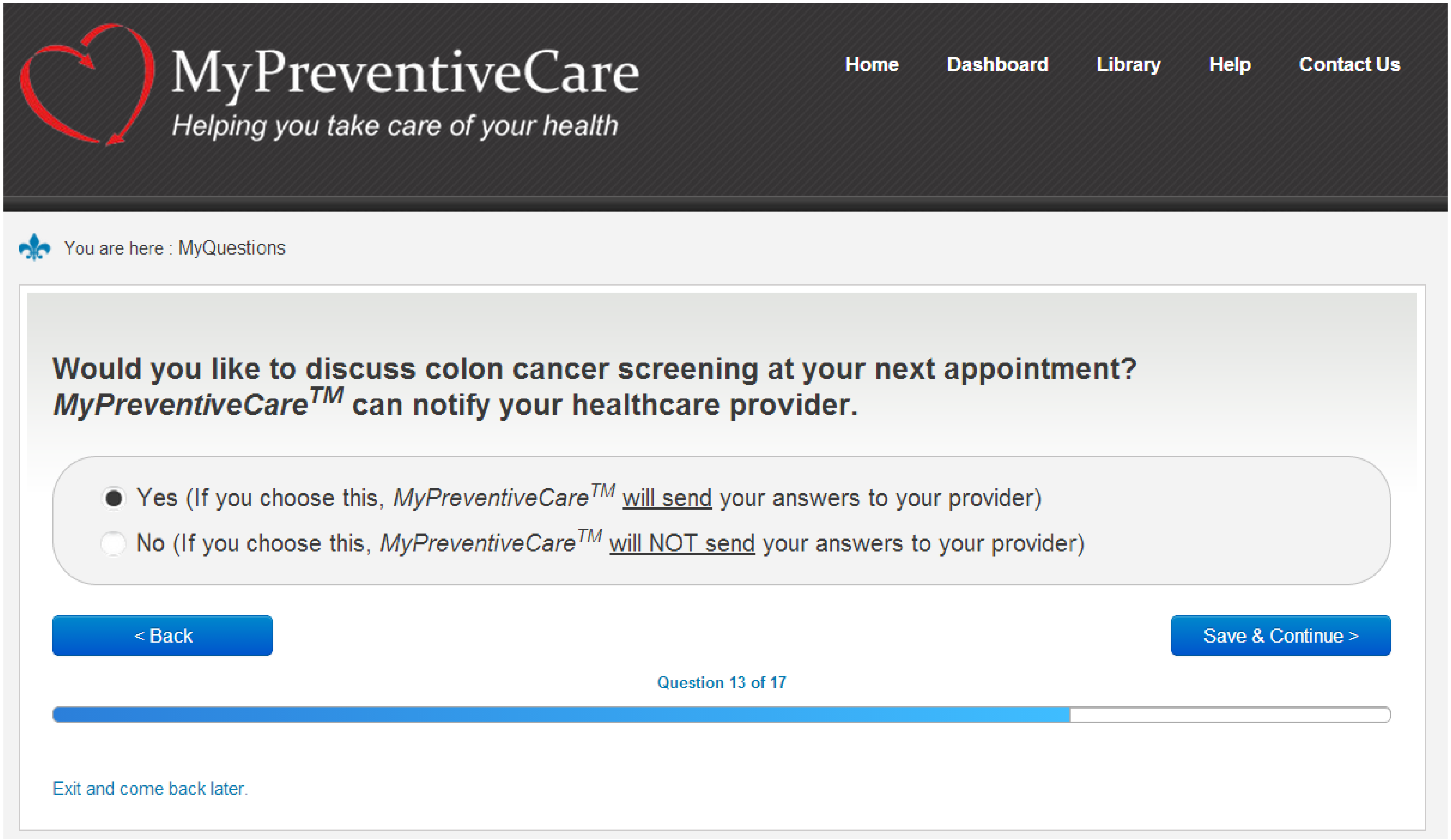

MyQuestions Question #14

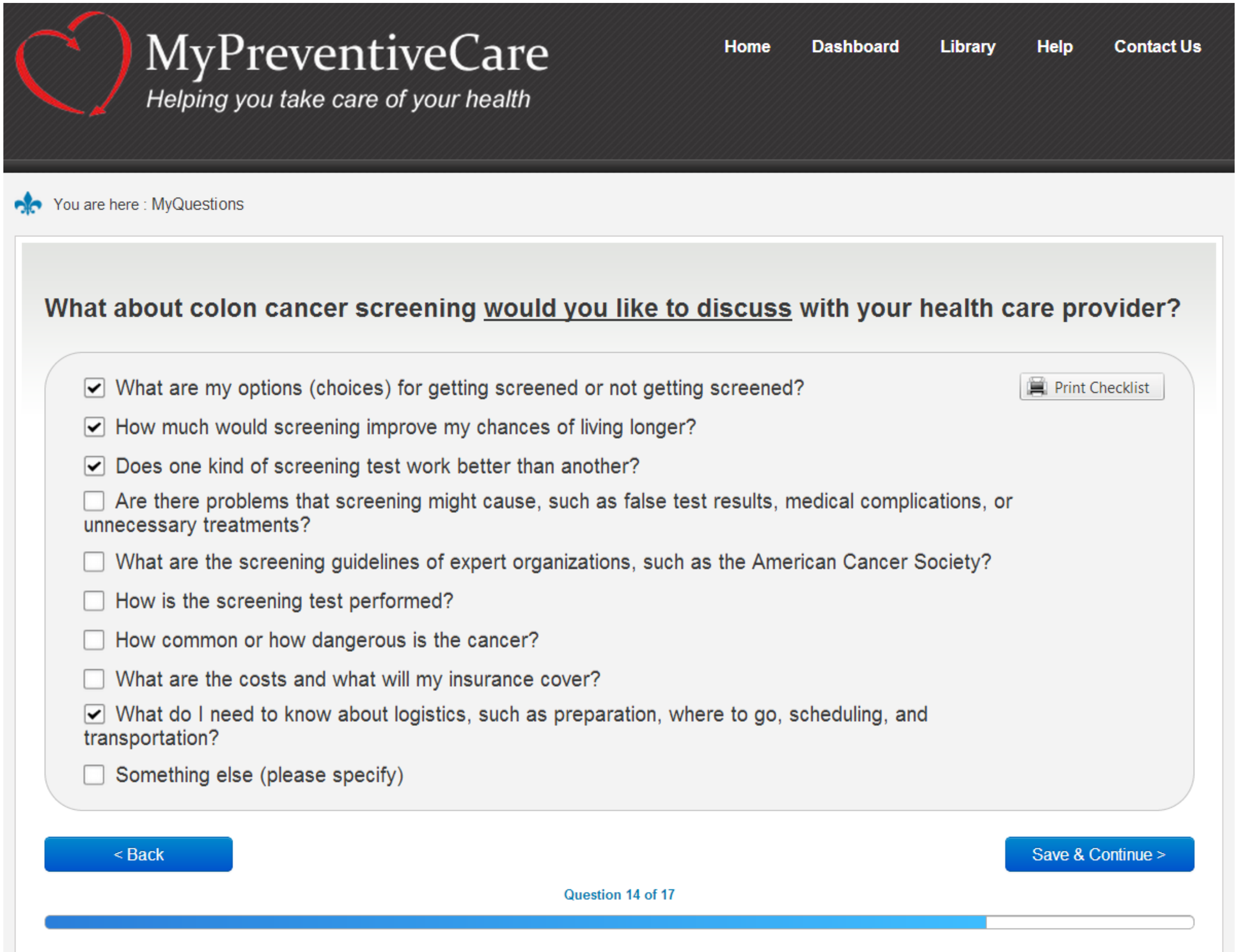

MyQuestions Question #15

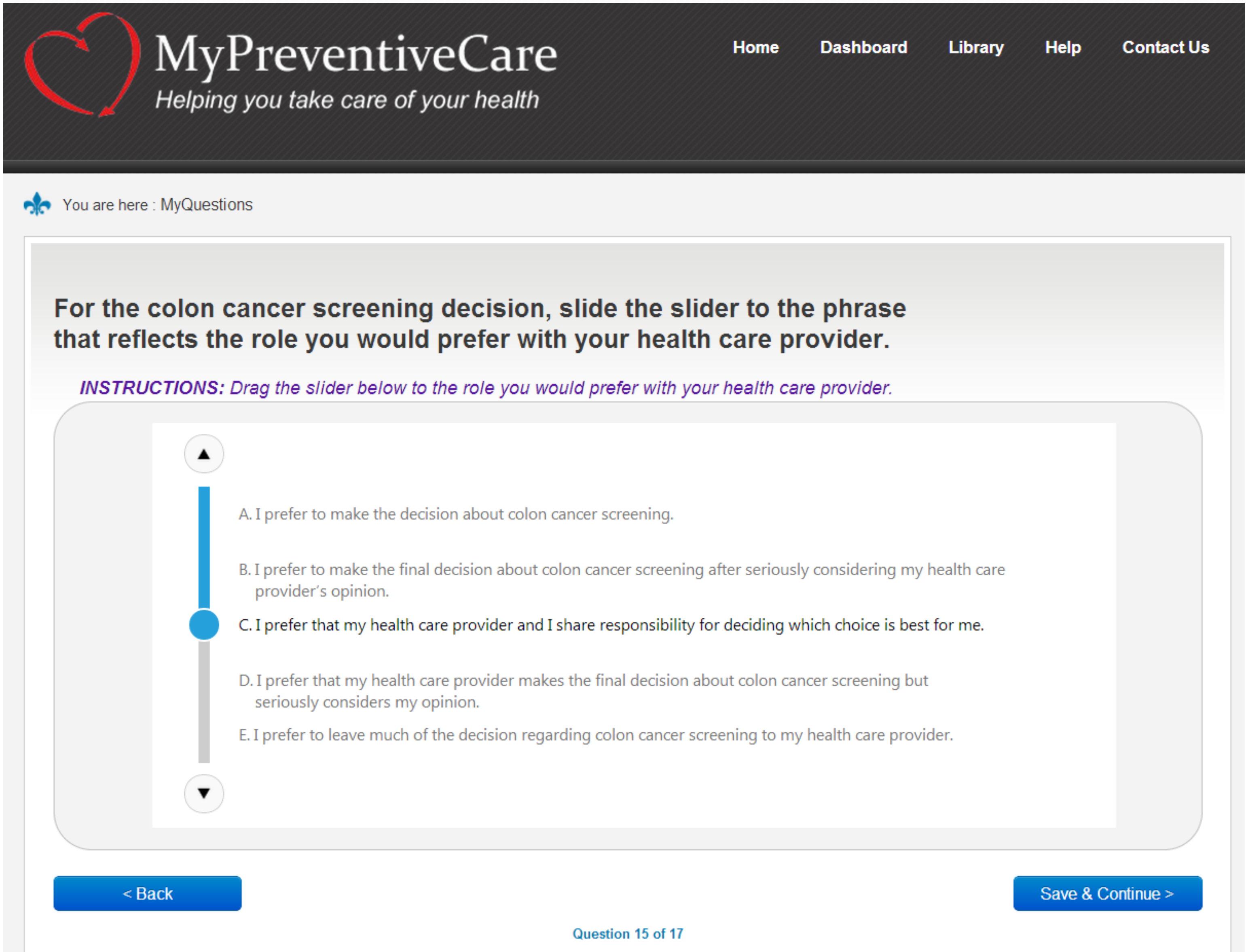

MyQuestions Question #16 – Invitation to have visit audio recorded

You are here : MyQuestions

Can you help us?

We want to see whether completing MyQuestions™ helps patients at their doctor's appointment. As part of a study, we are audio-recording about 30 appointments. All you would need to do is arrive a few minutes early for your next appointment to sign a consent form and allow your appointment to be recorded. In return we will thank you with a $50 Target gift card.

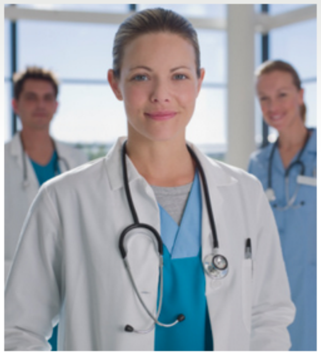

Want to learn more? Please enter your information and a coordinator will call with more details.

- (●) I am interested in learning more

Please enter your information and a coordinator will call you with more details.

Name ______

Number ______

Best time to contact me ______

- ( ) I am not interested in learning more at this time

MyQuestions transition page

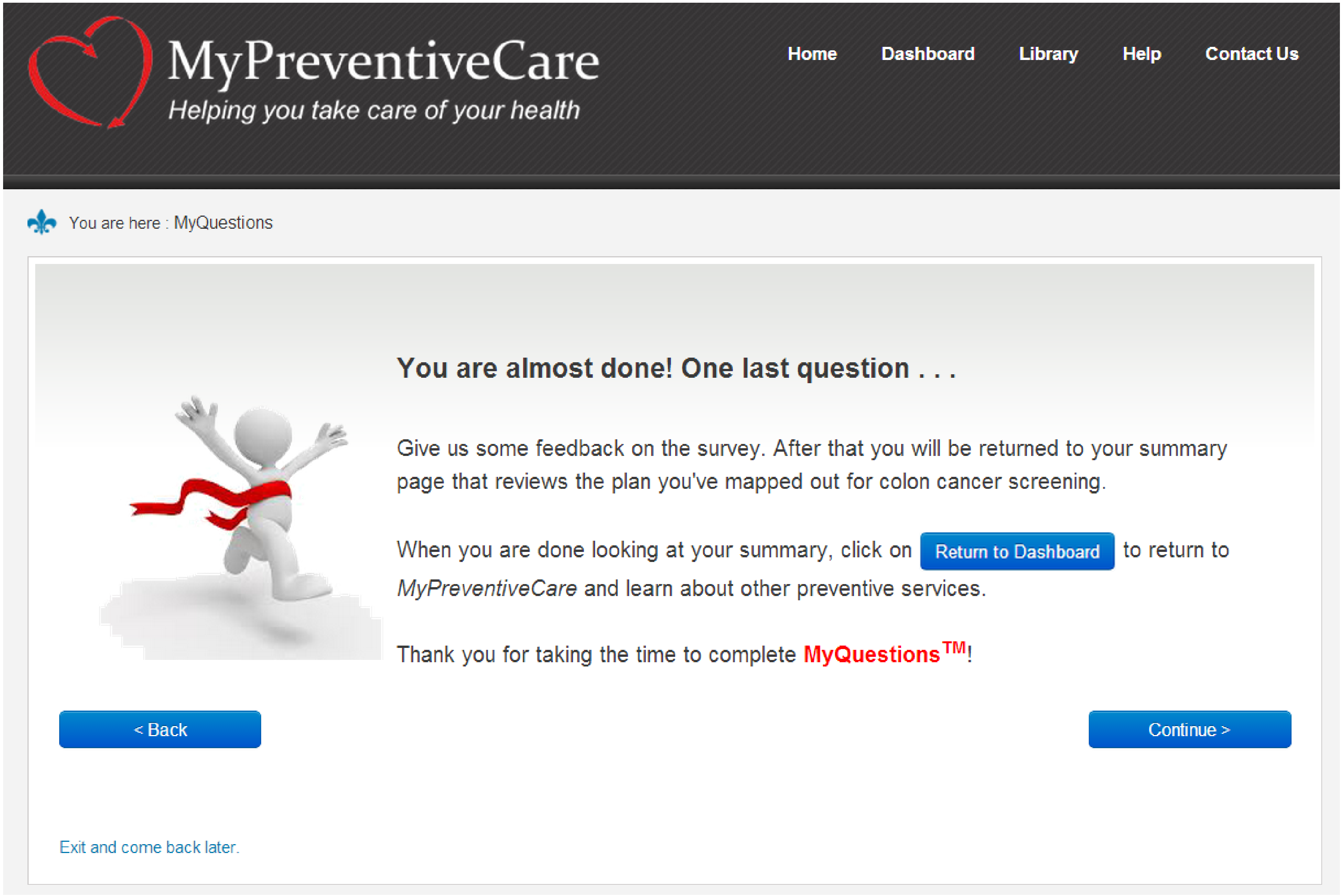

MyQuestions Question #17

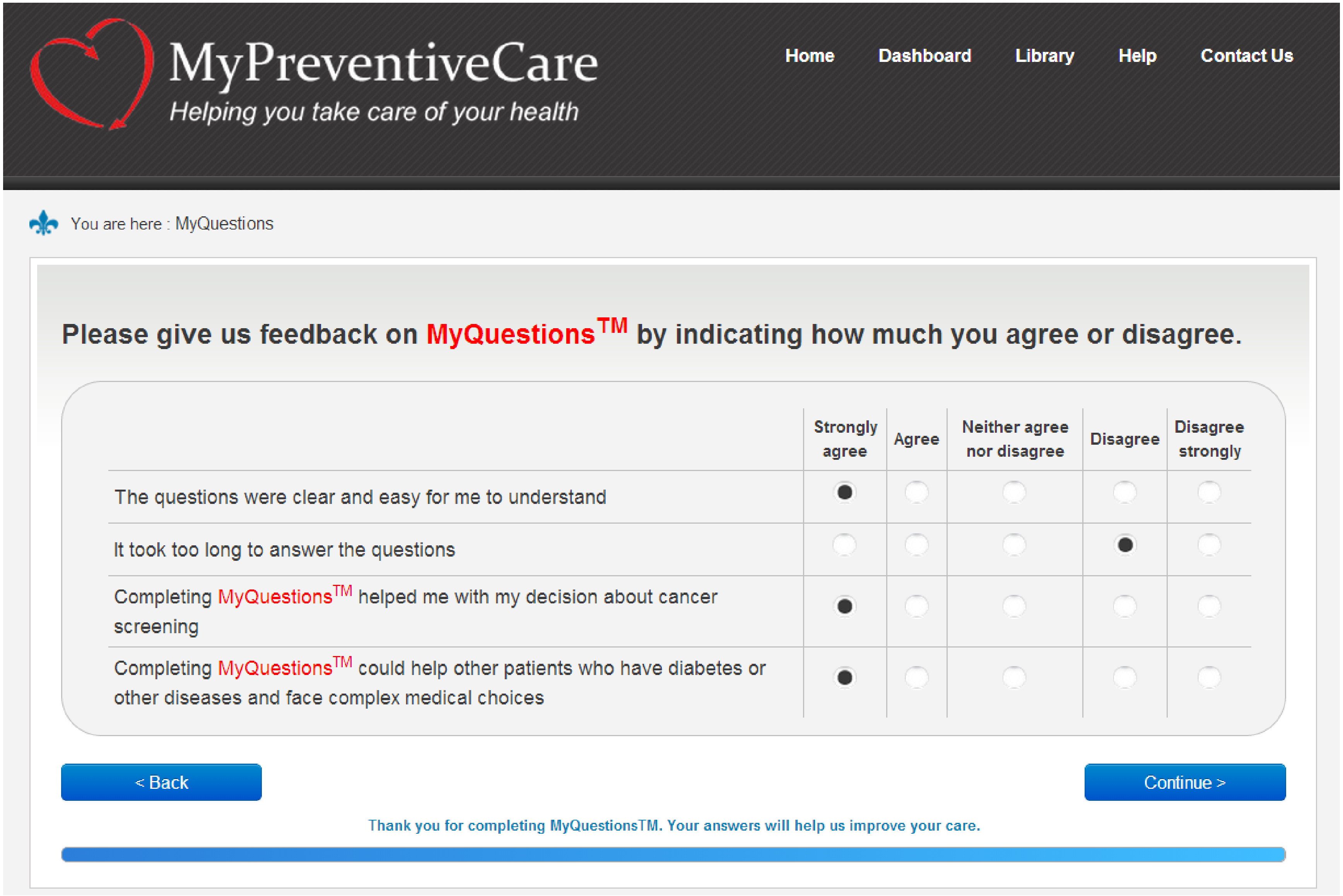

MyQuestions Action Page – created by patient responses to above questions

Get Tested for Colon Cancer

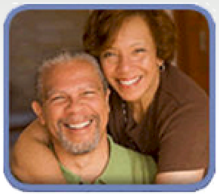

Your Information

You had a colonscopy on 2/27/2014.
Talk with your doctor about getting another colonoscopy, sigmoidoscopy, or home stool test now.

Your Action Plan

You've said you want to do the following:

Back to Dashboard
Print Action Plan

- I want to give it more thought.
- Get a reminder to make this decision
- Talk with my regular health care provider.
- Talk with my family, friends, clergy, or other people I trust.

You said you would like to notify your doctor that you want to discuss Colon Cancer Screening at your next visit and you specifically want to discuss:

- How much would screening improve my chances of living longer?
- What do I need to know about logistics, such as preparation, where to go, scheduling, and transportation?
- What are my options (choices) for getting screened or not getting screened?
- Does one kind of screening test work better than another?

You said that you would prefer that your health care provider and you share responsibility for deciding which choice is best for you.

You said you really wanted to know more about the following topics. **Click the tiles below to read more.**

Options for getting screened

Health benefits of screening

Problems that screening can cause

Costs and insurance coverage

More information about Colon Cancer Screening

The Basics

Men and women age 50 to 75 should be screened regularly for colon cancer. Any one of three tests can be used: a **colonoscopy** every 10 years, a **sigmoidoscopy** every 5 years, or a **home stool test** every 1 year.

The Benefits

If you act early, you can significantly reduce your chances of complications or death from colon cancer.

The Risks

Screening isn't perfect and can produce a "false-positive" result when there is no cancer. This can cause anxiety and lead to additional tests. Also, a colonoscopy can sometimes cause bleeding, damage to the colon, or other serious complications.

Example of summary of patient responses in the clinician's electronic health record

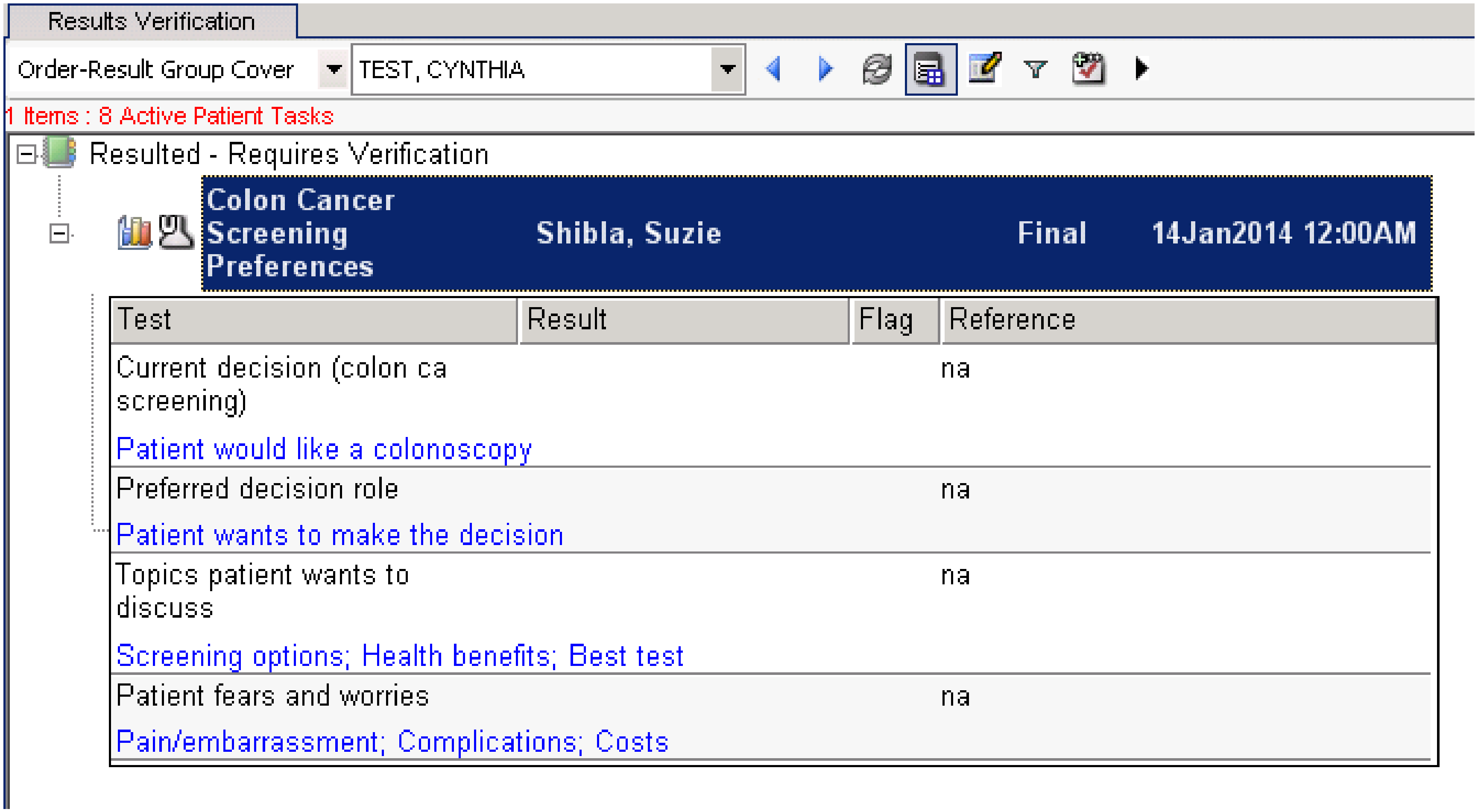

Test	Result	Flag	Reference
Current decision (colon ca screening)<br>Patient would like a colonoscopy			na
Preferred decision role<br>Patient wants to make the decision			na
Topics patient wants to discuss<br>Screening options; Health benefits; Best test			na
Patient fears and worries<br>Pain/embarrassment; Complications; Costs			na
